# Application of Next Generation Semiconductor-Based Sequencing for the Identification of *Apis mellifera Complementary Sex Determiner* (*csd*) Alleles from Honey DNA

**DOI:** 10.3390/insects12100868

**Published:** 2021-09-24

**Authors:** Samuele Bovo, Anisa Ribani, Valerio Joe Utzeri, Valeria Taurisano, Giuseppina Schiavo, Matteo Bolner, Luca Fontanesi

**Affiliations:** Department of Agricultural and Food Sciences, University of Bologna, Viale Giuseppe Fanin 46, 40127 Bologna, Italy; samuele.bovo@unibo.it (S.B.); anisa.ribani2@unibo.it (A.R.); valeriojoe.utzeri2@unibo.it (V.J.U.); valeria.taurisano2@unibo.it (V.T.); giuseppina.schiavo2@unibo.it (G.S.); matteo.bolner2@unibo.it (M.B.)

**Keywords:** environmental DNA, genetic diversity, honey bee, inbreeding, polymorphism, population genomics, variability

## Abstract

**Simple Summary:**

Honey contains traces of the DNA of the honey bees that produced it. This environmental DNA can therefore be used to investigate the genome of the honey bees. In this study, we used a next generation sequencing technology to analyze the variability of a key gene of *Apis mellifera* L., the *complementary sex determiner* (*csd*) gene, using honey environmental DNA as a source of honey bee DNA. This gene determines the sex of the bees. Two different alleles at this locus are needed to produce females whereas males have only one copy of this gene as they are haploid. In case two identical alleles are present in a diploid individual, the larvae are not vital and are discarded by the workers. Therefore, there is an advantage in maintaining a large *csd* diversity in honey bee populations. In light of the recent decline in honey bee populations, it is important to monitor the allele variability at this gene. The applied methodology provided a new strategy to disclose the genetic diversity at the *csd* gene at the population-wide level and identify most, if not all, *csd* alleles present in the colonies in a single analysis.

**Abstract:**

The *complementary sex determiner* (*csd*) gene plays an essential role in the sex determination of *Apis mellifera* L. Females develop only if fertilized eggs have functional heterozygous genotypes at this gene whereas males, being haploids, are hemizygous. Two identical *csd* alleles produce non vital males. In light of the recent decline in honey bee populations, it is therefore important to monitor the allele variability at this gene. In this study, we tested the application of next generation semiconductor-based sequencing technology (Ion Torrent) coupled with environmental honey DNA as a source of honey bee genome information to retrieve massive sequencing data for the analysis of variability at the hypervariable region (HVR) of the *csd* gene. DNA was extracted from 12 honey samples collected from honeycombs directly retrieved from 12 different colonies. A specifically designed bioinformatic pipeline, applied to analyze a total of about 1.5 million reads, identified a total of 160 different *csd* alleles, 55% of which were novel. The average number of alleles per sample was compatible with the number of expected patrilines per colony, according to the mating behavior of the queens. Allele diversity at the *csd* could also provide information useful to reconstruct the history of the honey.

## 1. Introduction

Honey bees (*Apis mellifera* L., 1758) are haplodiploid organisms like all species of the insect order Hymenoptera [1]. In these organisms, females develop from fertilized oocytes, which form diploid embryos, whereas males develop from unfertilized eggs, which produce haploid embryos [1,2]. This sex determination mechanism can work if females are heterozygous at a key genetic factor, the *complementary sex determiner* (*csd*) gene which, in this genotype condition, drives the sex determination cascade essential to produce female bees [3]. Drones, which are haploid, have only one copy of the *csd* gene and are defined as hemizygous. In diploid honey bees, if two functional identical *csd* alleles are present (i.e., homozygosity), the condition results in being similar to hemizygosity and the honey bees develop into sterile diploid male forms [3,4,5]. These diploid males are eliminated by the worker bees within three days of hatching as they would constitute a drain in colony resources and energy [6,7]. These eliminated larvae, however, cause decreased brood viability and, in turn, can affect colony growth and productivity due to the lack of worker bees [8,9,10].

The natural queen mating system, which involves polyandry and mating flight far from the original hives, decreases the risk of co-occurrence of identical *csd* alleles in fertilized eggs [10,11]. The frequency of identical *csd* alleles in diploid genomes could, however, increase with an increased level of inbreeding in honey bee populations, which can occur in close populations under selective breeding programs [6,8,9,12,13,14]. Therefore, the maintenance of a high allelic diversity at the *csd* gene is an important factor for the survival of honey bee populations, and several modeling systems have investigated the related genetic mechanism before and after this gene was recognized as the sex determination locus [15,16,17,18,19,20,21,22]. The number and distribution of *csd* alleles is particularly relevant in light of the problems that a reduction in the viability of the colonies could have in the context of honey bee population decline [23].

The *csd* gene is constituted by nine exons [3]. Exons 6, 7, and 8 encode the potential specifying domain that is under balancing selection [20,22]. Most of the allelic variation of the *csd* gene resides in the hypervariable region (HVR) encoded by exon 8 [3,21,23,24,25,26,27]. The HVR, flanked by conserved regions at both ends, consists of a variable number of A/T-rich nucleotide repeats, which mainly encode for a variable number of asparagine (N) and tyrosine (Y) residues. This repeat-rich region has a very high mutation rate, estimated to be higher than twice that of microsatellite regions, which can frequently result in the generation of new alleles [21].

The level of diversity of this highly repetitive gene region has been investigated in different honey bee populations by several studies that mostly used Sanger sequencing of the HVR amplified from drones [21,23,24,25,26,28,29]. These studies identified a high number of *csd* protein allele sequences that have been recently compiled in a scientific note [27].

Honey is a unique source of environmental DNA (eDNA) as it contains traces of all the organisms that directly or indirectly contributed to produce it or were part of the hive environment where it was produced [30,31,32,33,34,35,36,37,38]. Therefore, honey also contains the DNA of the honey bees that produced it [33]. We recently analyzed honey bee mitochondrial DNA (mtDNA) variability using honey as a source of DNA [33] and produced a distribution map of the main *A. mellifera* mtDNA lineages in Italy [34]. We also applied whole DNA shotgun sequencing approaches using next generation sequencing platforms for metagenomic analyses of honey DNA, and established a method to identify the *A. mellifera* subspecies using variants sequenced and retrieved from this matrix [31,32]. The method offers the possibility of analyzing more than one colony and many queen lineages at the same time, considering that honey (as it is usually prepared by the beekeepers) is derived from several families or even more than one apiary [33,34,37,38]. Therefore, honey can be used to obtain a quite extensive population-wide picture of the presence of honey bee genetic features including information on variability at the *csd* gene. Kolics et al. [29] already tested the possibility of obtaining sequence information of the *csd* HVR using amplicons produced from honey DNA and Illumina next generation sequencing.

Next generation semiconductor-based sequencing technology (i.e., the Ion Torrent platform) could provide a convenient high-throughput DNA sequencing system for routine applications due to the possibility of sequencing amplicons that are not obtained with specific adaptor primers, which could be relevant for target amplification from highly degraded DNA such as the DNA that can be extracted from the honey [36,37,38] or other processed food products including meat and dairy products [39,40]. On the other hand, sequencing data from this technology should be appropriately analyzed to overcome the problem derived by the homopolymeric regions, which increase the sequencing error rate [39,40].

In this study, we tested the possibility to analyze the *csd* HVR from honey DNA by using a next generation semiconductor-based sequencing platform combined with a specifically designed bioinformatic pipeline that was able to retrieve highly reliable protein deduced *csd* alleles from this targeted region.

## 2. Materials and Methods

### 2.1. Honey Samples and DNA Extraction

We analyzed twelve polyfloral honey samples (hereafter referred to as H1–H12) produced by nine different beekeepers in 2020 from twelve different apiaries located in five different provinces of the Emilia-Romagna region, north of Italy (Table 1). Honey samples were collected directly from one honeycomb retrieved from 12 different colonies. Seven different samples/apiaries were linked to seven different beekeepers, three other different samples/apiaries were linked to another beekeeper, and the last two samples/apiaries were linked to another different beekeeper (Table 1).

These samples were used for DNA extraction, following the protocol previously described [30,31,32,33]. Briefly, honey samples were pre-treated by adding ultrapure water in 50 g of starting material divided into four aliquots of 12.5 g. After vortexing and incubating at 40 °C for 1 min, the tubes were centrifuged at 5000× *g* at room temperature for 25 min. The resulting supernatant was eliminated and 5 mL of ultrapure water was added in each tube and then the content of the four tubes was merged in a single 50 mL tube. A second centrifugation at 5000× *g* for 25 min at room temperature followed and the supernatant was discarded. The resulting pellet was resuspended in 0.5 mL of ultrapure water and transferred in a 1.5 mL tube containing about 12 glass beads (500 µm) and vortexed for 3 min. The sample was then transferred in a new 1.5 mL tube removing the beads and stored at 4 °C. DNA extraction was performed using 1 mL of CTAB buffer [2% (*w/v*) cetyltrimethylammonium bromide; 1.4 M NaCl; 100mM Tris-HCl; 20 mM EDTA; pH 8.0], with the addition of 5 µL of RNase A solution (10 mg/mL) and 30 µL of proteinase K solution (20 mg/mL). Tubes were then incubated at 65 °C for 90 min after gently mixing, and centrifuged for 10 min at 16,000× *g*. A total of 700 µL of the obtained supernatant was transferred into a new tube containing 500 µL of chloroform/isoamyl alcohol (24:1) solution, vortexed for 30 s and then centrifuged at 16,000× *g* for 15 min at room temperature. The supernatant was transferred in a new 1.5 mL tube and the DNA was isolated and purified in two steps with isopropanol and then ethanol 70%. DNA was finally resuspended with 30 µL of sterile water and stored at −20 °C.

Extracted DNA was quality checked in a TBE 1% agarose gel after staining with 1× GelRed Nucleic Acid Gel Stain (Biotium Inc., Hayward, CA, USA) and the concentration was measured using a Qubit 2.0 fluorimeter (Thermo Fisher Scientific, Waltham, MA, USA). This quality control analysis showed that the extracted DNA from all honey samples was degraded, as expected, confirming previous evaluations [33,36,37,38].

### 2.2. PCR Amplification of the csd Region

The *csd* HVR was amplified using a primer pair reported by Hyink et al. [28], also used by Zareba et al. [23]: forward: 5′-TATCGAGAAAsATCGAAAGAACGAT-3′, reverse: 5′-ATTGAAATCCAAGGTCCCATTGGT-3′. Amplifications were performed on a 2700 Thermal Cycler (Life Technologies, Waltham, MA, USA). Reactions were run in a total volume of 20 μL including KAPA HiFi HotStart Mastermix (Roche, Basel, Switzerland); 10 pmol of each primer; 40 ng of template DNA. The PCR profile was the following: initial denaturation step at 95 °C for 3 min; 35 cycles of alternate temperatures (20 s at 98 °C, 15 s at 51 °C, 30 s at 72 °C); and a final extension step at 72 °C for 1 min. Obtained amplicons were electrophoresed on 2.5% agarose gels in TBE 1× buffer and then visualized with 1× GelRed Nucleic Acid Gel Stain (Biotium Inc., Hayward, CA, USA).

### 2.3. Next Generation Sequencing

Sequencing of the obtained amplicons was carried out following the protocol already described [41] with a few modifications. Briefly, PCR products obtained from each honey DNA sample using *csd* primers were purified with ExoSAP-IT^®^ (USB Corporation, Cleveland, OH, USA) and then sequenced using an Ion S5-Ion Chef System (Thermo Fisher Scientific Inc., Waltham, MA, USA). A total of 12 libraries were produced by end-repair and ligation of the DNA fragments with a specific barcode using the Ion Xpress^TM^ Plus Fragment Library and Ion Xpress™ Barcode Adapter 1–32 kits (Thermo Fisher Scientific Inc.). Each library was quantified with the Ion Library TaqMan Quantitation Kit (Thermo Fisher Scientific Inc.) by qPCR with the QuantStudio™ 7 Pro Real-Time PCR System (Thermo Fisher Scientific Inc.). Libraries were first clonally amplified by emulsion PCR and sequenced following the manufacturer’s instructions using the Ion 510™ and Ion 520™ and Ion 530™ Kit-Chef after having pooled them for sequencing in one Ion 520 chip (Thermo Fisher Scientific Inc.).

### 2.4. Bioinformatic and Data Analyses

#### 2.4.1. Read Filtering and Identification of *csd* Sequences

Reads were pre-processed with the Torrent Suite v.5.8.0 (Thermo Fisher Scientific Inc.) and a fastq file was obtained for each barcode. Extraction of reads covering the *csd* HVR relied on the identification of nucleotide sequence coding for the highly conserved protein residues (motif) upstream (e.g., KIIS) and downstream (e.g., IEQIP) of the repetitive region [27]. Protein motives were inferred from about 500 protein sequences covering the *csd* HVR available in UniprotKB [42] (accessed on 2 August 2021) and aligned with MAFFT [43]. This allowed us to generate two regular expressions (one for the 5′ and 3′ HVR) specifying the DNA based search patterns used to interrogate reads (both in forward and in reverse complement). Trimming at the level of these two patterns was carried out and only trimmed reads with a Phred quality score (Q) greater or equal to six (probability of incorrect base call less than 25%) at each nucleotide position were retained and exported as a fastq file. Moreover, to filter out additional low-quality reads, DNA sequences were clustered together and clusters presenting less than 10 reads were discarded. The obtained smaller fastq files were quality checked using the *fastqc* tool (http://www.bioinformatics.bbsrc.ac.uk/projects/fastqc/; accessed on 2 August 2021), which points out high quality sequences. Finally, to obtain the *csd* alleles, reads were translated to small peptides. All analyses were implemented in Python 2.7.

#### 2.4.2. Quality Control of *csd* Alleles

Obtained protein sequences were considered functional *csd* alleles and were retained for further analyses if they satisfied the following criteria: (i) presence of both HVR flanking protein motives and absence of any stop codon (indels would disrupt the coding frame); (ii) alleles detected in only one sample with a relative frequency (within sample) of reads coding for the defined allele greater than 0.0164, which represents the median minimum relative abundance of the alleles detected in more than one sample; and (iii) alleles detected in at least two honey samples, irrespective of their read abundance. Rarefaction curves were used to evaluate the sequencing efforts in terms of captured variability. For each sample, the average number of functional alleles was plotted as a function of the percentage of sequenced reads, randomly sampled (without replacement). One hundred different sets of sampled reads were used to compute the average number of functional alleles. Analyses were implemented in Python 2.7.

#### 2.4.3. Evaluation of *csd* Polymorphims

The full set of functional alleles was imported in Jalview v.2.11.1.3 [44], redundancy was removed, and pairwise alignments were performed using the default alignment parameters. For each alignment (pair of alleles), differences were evaluated as proposed by Lechner et al. [21] and also applied by Zareba et al. [24] by using an index that considered the difference in the HVR length (ΔL_HVR_), which was summed to the number of amino acid substitutions (N_SAP_; non identical residues). A multiple sequence alignment of functional alleles was obtained by using MAFFT [43]. Analyses were also carried out within the honey sample based on the specific allelic set. R v.3.6.0: [45] was used to elaborate data and generate figures. A BLASTP search (https://blast.ncbi.nlm.nih.gov/Blast.cgi; accessed on August 2021) was carried out to classify alleles as novel.

#### 2.4.4. Evaluation of Honey Sample Similarity

Similarity of the analyzed honey samples was investigated via a principal component analysis (PCA) on a binary data matrix accounting for the presence (1) or absence (0) of a *csd* allele. The matrix had size = *n* × *m*; where *n* was the number of samples whereas *m* was the number of non-identical alleles identified in the whole dataset of honey samples. To carefully handle this data type, logistic PCA was applied [46]. Moreover, based on this matrix, we also evaluated sample diversity by means of the Jaccard index (J). A dissimilarity matrix (1-J) was computed and hierarchical clustering was applied to it. As a clustering method, we applied both single and complete linkage. Finally, multidimensional scaling (MDS) was applied to the dissimilarity matrix. Analysis was carried out in R v.3.6.0 (package *logisticPCA*; functions *cv.lpca*, *logpca_model*, *dist*, and *cmdscale*).

## 3. Results

### 3.1. Sequenced Reads and Identified csd Alleles

Table 1 summarizes the sequencing statistics and the identified *csd* alleles from the analyzed honey samples. The amplicon-based semiconductor sequencing of the targeted *csd* gene region produced a total of 1,524,796 reads. Read counts ranged from 1341 to 414,002 for H12 and H3, respectively. Reads were then translated and filtered according to the presence of the 5′ (e.g., KIIS) and 3′ (e.g., IEQIP) amino acid motifs, the highly conserved HVR flanking regions [20,21,23,27], and the absence of stop codons. The proportion of these reads coding for retained protein alleles over all reads was 75.3% and ranged from 48.3% (H7) to 97.5% (H6). H10 was the sample with the highest number of protein alleles (*n* = 61) and H11 and H12 were the samples that had the lowest number (*n* = 10). Table S1 reports the complete list of all alleles identified from each sample with their relative abundance. The Pearson’s correlation coefficient (*r*) between the number of sequenced reads or the number of retained reads per sample and the number of putative functionally different protein alleles per sample was low (*r* = 0.28, *p* = 0.37 and *r* = 0.22, *p* = 0.48, respectively). Considering all the honey samples, we identified a total of 160 unique *csd* protein alleles (Table S2), of which 114 (71.25%) were private alleles (i.e., detected in only one honey sample). Figure S1 shows the distribution (presence/absence) across samples of these 160 alleles. Rarefaction curves (Figure S2) pointed out that all the possible alleles were detected as curves quickly reached a plateau. In fact, it was possible to observe that the whole set of alleles specific to each sample was detected when less than the 30% of the reads were sampled (range 14–29%). The number of private alleles per sample ranged from two (H4 and H11) to 41 (H10) and was highly correlated with the total number of alleles identified per sample (*r* = 0.97, *p* = 3.6 × 10^−7^). Of the 160 unique alleles, 88 were novel (i.e., not yet reported in GenBank). The largest number of novel alleles was identified in H10 (*n* = 40), which was also the sample with the largest number of all alleles (*n* = 61) and of private alleles (*n* = 41). H12 did not have any novel alleles as all 10 alleles identified from this sample were also already deposited in GenBank. The number of novel functional alleles identified per sample was highly correlated with the total number of identified alleles per sample (*r* = 0.95; *p* = 2.3 × 10^−6^), but was not correlated with the number of sequenced reads per sample or the number of functional retained reads per sample (*r* = 0.04, *p* = 0.89 and *r* = 0.041, *p* = 0.89, respectively).

The list of *csd* protein alleles identified in the different samples with a relative abundance >5% is reported in Table 2. Honey samples had from one (H6 and H10) to five (H12) abundant alleles. On average, 3 ± 1 alleles with at least this abundance were detected in each honey sample. Among the most abundant alleles, only four were novel alleles, detected in three honey samples (H3, H7, and H9; Table 2). Some alleles were identified in more than one sample: one allele was identified in two different samples (H1 and H2); three alleles were identified in three different samples (H2, H10, and H12; H5, H7, and H11; H5, H8, and H11); one allele was identified in five samples (H2, H4, H6, H8, and H11).

### 3.2. Diversity of csd Protein Alleles

We then investigated the level of genetic diversity at the *csd* gene considering all of the analyzed samples together (thereafter indicted as population-wide analysis) or considering only data within each sample separately. For this specific purpose, we used the HVR length (ΔL_HVR_) and the number of amino acid substitutions (N_SAP_) between all allele pairs.

**Figure 1:**
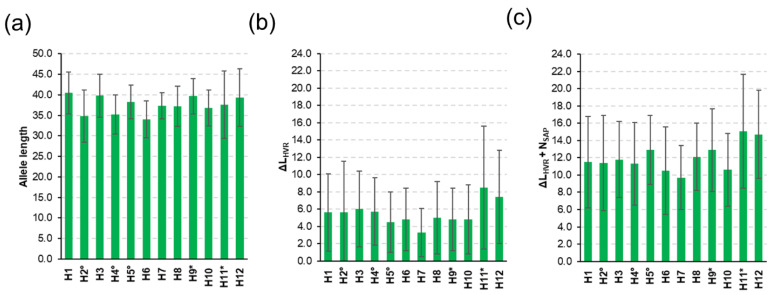
Study of the within sample HVR *csd* variability. (**a**) Allele length (mean ± s.d.); (**b**) ΔL_HVR_ (mean ± s.d.); (**c**) Total differences (ΔL_HVR_ + N_SAP_; mean ± s.d.). ° Samples belonging to the same beekeeper. * Samples belonging to the same beekeeper.

The HVR length ranged from 35 to 53 residues, with a mean ± standard deviation of 38 ± 5 (median = 37). No relevant deviations emerged in the analysis of the single samples (Table S3 and Figure 1a). Based on pairwise comparisons, two alleles had on average a ΔL_HVR_ = 5 (s.d. = 4). Within samples, ΔL_HVR_ values ranged from 3.33 ± 2.83 (H7) to 8.47 ± 7.07 (H11). Considering the median values, ΔL_HVR_ ranged from three (H2 and H7) to seven (H12). Details are provided in Table S3 and Figure 1b.

**Figure 2:**
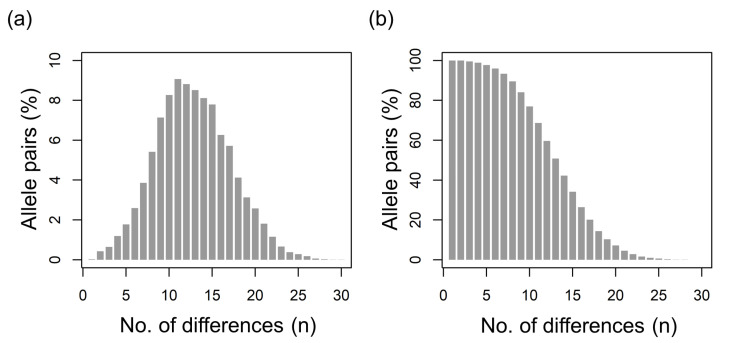
Study of the HVR *csd* variability via pairwise alignment of 160 non-identical alleles identified in the investigated honey samples. Differences considered the ΔL_HVR_ + N_SAP_ values. (**a**) Distribution of the HVR differences (alignments presenting *n* differences). (**b**) Cumulative distribution of the HVR differences (% of alignments presenting ≥ *n* differences).

Out of 12,720 pairwise alignments (Figure 2), the global sequence diversity (ΔL_HVR_ + N_SAP_) ranged from 0 (3 alignments; 0.02%) to 29 (1 alignment; 0.008%). On average, 12 differences (s.d. = 4, median = mean) characterized the 160 alleles. The samples with the lowest and highest pairwise maximum diversity were H4 (max = 19) and H12 (max = 29), respectively. The averaged diversity (ΔL_HVR_ + N_SAP_) ranged from 9.7 (H7) to 15.1 (H11). Considering the median ΔL_HVR_ + N_SAP_ values, sample H7 had the smallest (*n* = 10) and samples H11 and H12 had the largest (n. = 14) number of differences, the details of which are included in Table S3 and Figure 1c. It was interesting to note that the honey samples (H11 and H12) that had the lowest number of *csd* protein alleles (*n* = 10) had also the highest averaged diversity values (15.1 and 14.7, respectively) and that there was a low negative correlation across all samples between the number of alleles and the average number of sequence diversity (*r* = −0.35; *p* = 0.26). Figure S3 shows the multiple sequence alignment of the 160 protein alleles.

### 3.3. Honey Sample Similarity

**Figure 3:**
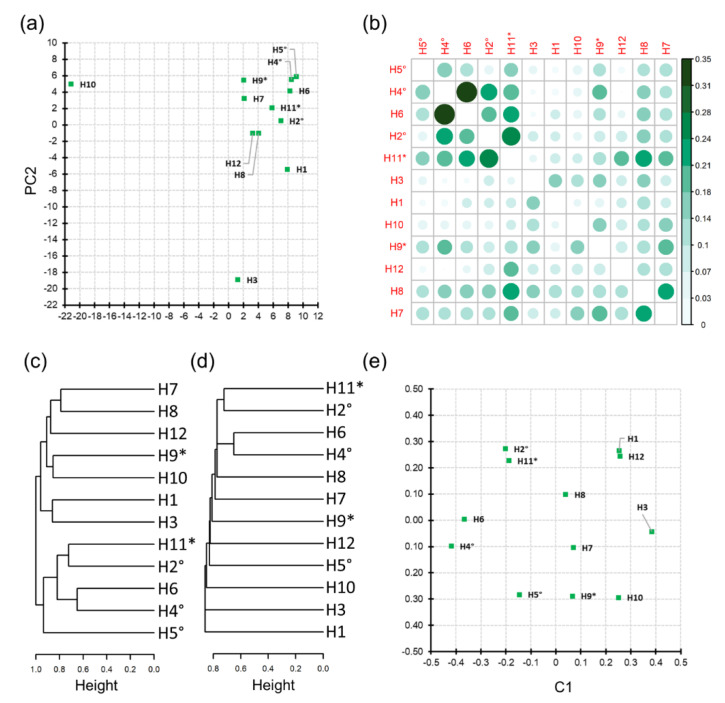
Analyses of honey sample similarity: (**a**) logistic PCA; (**b**) Jaccard similarity coefficient; (**c**) single-linkage clustering based on Jaccard distance; (**d**) complete-linkage clustering based on Jaccard distance; (**e**) multidimensional scaling of the Jaccard distance matrix. Samples provided by the same beekeeper are marked with the same symbol (* or °).

Similarity between honey samples obtained from the distribution (presence/absence) of the identified *csd* alleles was initially evaluated via logistic PCA. This analysis, considering the global properties of the dataset, pointed out a major group of samples without any specific structure (Figure 3a). Two outlier samples emerged—H3 and H10—an expected result as those were the samples with the highest number of detected alleles, most of them being private. Samples H4 and H5, two out of three samples coming from the same beekeeper, showed close results to each other. Based on the Jaccard index and locally comparing pairs of samples (sample specific alleles), the similarities values ranged from 0 (H4 and H12) to 0.35 (H5 and H6). Samples H2, H4, and H5, belonging to the same beekeeper, did not present high similarity values (from J = 0.06 to J = 0.23). For the second set of honey samples (H9 and H11) derived from the same beekeeper, a low similarity index was retrieved (J = 0.13). Similarities are presented in Supplementary Table S4 and Figure 3b.

Clustering based on the complete linkage approach graphically evidenced the similarities presented via the J matrix, highlighting high similarity between samples H4 and H6 and between H2 and H11 (Figure 3c). Moreover, two main groups of samples emerged in this analysis, one of these comprising the three samples H2, H4, and H5 obtained from the same beekeeper. Single linkage hierarchical clustering confirmed the H4 and H6 and the H2 and H11 clusters (Figure 3d). This analysis also confirmed the higher dissimilarity of samples H1, H3, and H10 that emerged with the logistic PCA. The multidimensional scaling plot returned similar results, summarizing the similarity analyses in 2D (Figure 3e).

## 4. Discussion

The *csd* gene has a key role in sex determination in *A. mellifera* [3]. The analysis of the sequence variability in the HVR of this gene can provide information useful to monitor the level of inbreeding in a honey bee population [23,24,25,26,27,28]. It has also been suggested that *csd* may play a role in controlling the balance between inbreeding and outbreeding in honey bee reproduction [11,17,23]. Considering that heterozygosity at this gene is essential to generate vital females, several studies have investigated *csd* sequence diversity, demonstrating that a large number of functional alleles exist in all honey bee populations [21,23,24,25,26,27,28,29]. These studies have mainly been carried out by Sanger sequencing the HVR from drones of different colonies, in order to maximize the probability of detecting the two alleles of the queen bees [23,24,28] or from workers after cloning the amplicons [25]. The uneven distribution of many infrequent alleles suggests that the diversity of the *csd* gene is largely underestimated [23]. Therefore, it is necessary to further extend the sequence analysis of this gene to better understand the mechanisms that generate and spread different alleles and to better investigate the role of this locus in maintaining diversity in honey bee populations [23].

The use of next generation sequencing can increase the amount of sequencing data to a few orders of magnitude, which is useful for an extended analysis of the sequence diversity at targeted genes. In this study, we tested the application of next generation semiconductor-based sequencing technology (Ion Torrent/S5) coupled with honey eDNA as the source of honey bee genetic information to obtain massive sequencing data for the analysis of *csd* gene variability at the population level.

We successfully produced a large number of sequence information that we filtered to overcome some of the problems derived by the applied sequencing technology and by the matrix from which honey bee DNA was recovered. The errors generated by the sequencing technology were removed with a pipeline that considered read quality and the expected in-frame variability to produce functional proteins, flanked by specific residues. About 25% of the sequencing reads were discarded, leaving about 1.1 million useful reads that were subsequently analyzed to define the level of variability in the *csd* gene that was recovered from the investigated honey samples. A larger number of reads, which could eventually be possible to generate with this sequencing technology (using different chips and chemistry) or using other sequencing technologies (e.g., Illumina) would not be needed as there was a very poor correlation (*r* = 0.28) between the number of alleles and the number of reads per sample (*r* = 0.23, if we only considered the retained functional reads). This is also expected if we consider that a colony (from which the honey was obtained) has a finite number of *csd* alleles, which are derived from its genetic history: the number of drones that fertilized the queen of the colony from which the honey derived, the number of queens that were eventually part of the family history from which the honey was collected and the contribution of the drifted workers.

One of the problems that we could not completely control was the degradation of the DNA extracted from honey. DNA extracted from commercial honey is usually highly degraded as the food matrix environment and its preservation conditions are not optimal to maintain DNA. Therefore, from this matrix, it is usually possible to amplify, without any biases, short DNA fragments [33,36,37,38]. To minimize the problems derived by long preservations that would increase DNA degradation, we collected honey samples directly from honeycombs. The amplification of the HVR fragments (about 300–380 bp) was successful in all cases, suggesting that, despite the problem of DNA degradation, honey can be a useful source of DNA for *csd* sequence analyses. Some biases in the amplification, however, probably occurred, as deduced from the number of the most abundant alleles obtained from the analyzed samples (Table 2). In three samples (H4, H6, and H10), only one allele had a percentage of reads that was >5% and these alleles were among the shortest alleles that we considered in the analysis of abundance. This means that these alleles were probably favored in the amplification/sequencing steps, creating some biases. At least two highly prevalent alleles, derived by the two alleles of the queen and, for that reason, with similar abundance, would be expected. This would be true in theory, in the case of no biases in the amplification and sequencing processes. If we also consider all other honey samples, it is possible to note that only in four cases did the two prevalent alleles have a similar or close abundance (H2: 53.7 and 30.2%; H8: 33.3 and 25.9%; H11: 43.8 and 32.5%; H12: 37.5 and 31.5%). It is worth noting that for H12, which was the sample with the lowest number of sequenced reads, there was also the lowest differences in terms of abundance between the two most prevalent alleles, despite the fact that these two alleles were quite different in terms of size of the considered functional region (37 and 49 residues, respectively). Biases in the abundance of the two queen derived alleles have been also reported in a pilot study that sequenced the *csd* HVR fragment amplified from honey DNA using the Illumina technology [29]. Even if it seems clear that the results derived by these approaches could only be partially considered as semi-quantitative [30,39,40,41,47], it will be important to evaluate how it could be possible to reduce and then manage the different sources of biases that are derived from the sequencing technologies and the degradation of the honey DNA.

Despite the potential biases that we discussed above, the use of honey DNA as a source of honey bee *csd* sequence information offers the possibly to detect most, if not all, of the *csd* alleles present in a colony, opening new opportunities to investigate *csd* diversity at population-wide levels. The results we obtained confirmed the presence of many infrequent alleles in the *A. mellifera* population. On average, each sample had about 23 different alleles, which is close to the number of patrilines present in a family as estimated by microsatellite analyses [48,49]. We also have to consider that at least two of these detected alleles might be from the maternal lineage (i.e., queen). A study that analyzed *A. m. carnica* colonies in mainland mating apiaries reported an average number of effective males equal to 20 [48] and another study that investigated colonies from different *A. mellifera* subspecies reported a range of 7–20 patrilines [49]. The first mentioned study [48] also detected drifted workers from other families (<5%), which can contribute to an increase in the number of paternal alleles in a colony. In both microsatellite derived estimations [48,49] several paternal lineages were present at very low frequency, also matching the low frequency (or abundance in our case) of several alleles that we identified from honey. For a few honey samples (H3, H9, and H10; Table 1) that had a larger number of alleles, we could suppose a more complicated genetic history probably due to one or more of the following situations: the subsequent presence of different queens leading the colony; the exchange of the comb to more than one colony; and/or a high rate of drift or combinations of different nuclei. To verify these hypotheses, we are designing studies that will make it possible to verify the information retrieved from the honey with information directly obtained from the honey bees of that colony.

A large proportion of alleles (55%) were also newly detected in this study. We could also predict that more new alleles would be discovered if the number of analyzed samples is increased. Even if we could not completely exclude that few of these alleles are derived by sequencing errors (which we could not completely eliminate, as in all sequencing experiments), clearly the large number of *csd* alleles may raise some questions (i) on the population dynamics and spreading or extinction of these alleles; (ii) on the mechanisms of generation of new alleles; (iii) on their role in defining a balance between inbreeding/outbreeding in the honey bee populations [23] and; in turn, (iv) on the usefulness of this locus in estimating and monitoring genetic diversity in *A. mellifera*. Additional studies are needed to clarify these issues and the approach that we tested here (i.e., massive sequencing from honey DNA) could help to answer, at least in part, some of these questions, if applied to larger scales and in appropriately planned experimental designs.

The analysis of putative functional allele diversity based on the number of amino acid substitutions and allele length differences (ΔL_HVR_ + N_SAP_) obtained a pairwise distribution of difference almost identical to that also reported in Polish honey bee populations that used Sanger sequencing of the *csd* HVR [23]. The quite high level of pairwise allele diversity (on average: 12 differences) was a little bit lower than that reported in the Polish populations (on average: about 14–15 differences [23]). This difference could be probably due to the lower range of differences that we observed (0 to 29) than what was reported by [23] (1 to 36). The reasons for this difference could be attributed (i) to the lower number of large alleles that we could amplify/sequence from the degraded honey DNA (as discussed above, a potential bias in our study) than what could be obtained directly from the drone DNA in [23]; (ii) to a higher level of functional inbreeding in the Italian population analyzed in our study (from a limited number of colonies) than in the Polish populations that were also investigated from a larger number of colonies [23]; and/or (iii) to different levels of subspecies introgression (which usually increases variability) between the Italian and the Polish honey bee populations. This latter aspect could be hypothesized considering the high rate of hybridization of the original dark bee in Poland, as demonstrated by the mitochondrial and nuclear DNA markers [50], and the relatively lower level of hybridization that might have experienced the *A. m. ligustica*, particularly in the Italian region from which the analyzed samples originated (Emilia-Romagna), as could be inferred from our recent mtDNA investigation [34]. Other studies are needed to better address these questions.

All honey samples that we investigated could be easily differentiated using the *csd* allele information. Even if it is not completely appropriate, we could extend this clear differentiation to all families from which the honey is derived. In fact, honey does not only contain DNA traces from the family from which it was sampled (in general, the most important source of honey bee DNA), but also provides hints from the whole history that it had including the fingerprinting of more than one queen (in the case of queen substitution), drifted individuals, the subsequent use or re-use of the comb, which all together may have contributed to distinguish their *csd* profiles. Therefore, it is possible to propose the use of the *csd* sequence variability as a potential tool to trace and authenticate the origin of the honey and, if applied to the honey bees, as a simple genetic footprint of the colony that produced it. Honey samples obtained from different apiaries of the same beekeeper were more similar in terms of *csd* profile only in one case (H4 and H5). This means that these samples were probably derived from queens of the same genetic line. It was not possible, however, to trace back the genetic information of the queens as the beekeeper did not record any data. Genomic analyses of the honey bees sampled from the same colonies will provide additional information to support what obtained from the honey-derived *csd* gene sequences. All remaining honey samples could not be grouped according to beekeeper origin, suggesting that quite a large heterogeneity was present in the colonies and apiaries that provided the honey samples.

## 5. Conclusions

Environmental DNA contained in the honey can be analyzed for many different purposes. In this study, we further expanded the usefulness of honey eDNA by targeting honey bee nuclear DNA to investigate, using a next generation sequencing technology, the variability of the key gene for sex determination in *A. mellifera*. Technical issues can be managed and considered to correctly interpret the final results that were, in general, in agreement with those reported by conventional Sanger sequencing approaches based on individual bee analyses. The tested approach, however, has the possibility of extending the amount of information that is needed to understand, from a population genetic perspective, all the open questions derived by this hypervariable locus. Other studies are needed to complete the analysis of the potential biases that the combined use of next generation sequencing and honey DNA can introduce in this context. As a general outlook, it will be possible in the future to use *csd* information retrieved from the honey to implement breeding plans in honey bees that would need data on the genetic closeness of the families, which might be determined by the number and differences of the *csd* alleles they carry.

## Figures and Tables

**Table 1:** Honey samples and their geographic origin (province), number of sequenced reads, and *csd* alleles identified from the sequencing data.

Sample ID ^1^	Province	No. of Reads	No. of Retained Reads (%) ^2^	No. of *csd* Protein Alleles ^3^	No. of Private *csd* Alleles (%) ^4^	No. of New *csd* Alleles (Private) ^5^
H1	Bologna	173,064	149,678 (86.4)	17	8 (47.1)	9 (7)
H2 °	Reggio Emilia	75,454	72,027 (95.4)	13	4 (30.8)	5 (3)
H3	Piacenza	414,002	269,785 (64.8)	40	16 (40.0)	13 (11)
H4 °	Reggio Emilia	84,307	82,056 (97.3)	14	2 (14.3)	5 (0)
H5 °	Reggio Emilia	397,333	296,982 (74.7)	20	7 (35.0)	5 (3)
H6	Ferrara	34,350	33,503 (97.5)	13	3 (23.1)	7 (3)
H7	Piacenza	99,979	48,369 (48.3)	23	11 (47.8)	12 (11)
H8	Bologna	41,741	37,431 (89.7)	17	4 (23.5)	3 (1)
H9 *	Piacenza	97,735	73,994 (75.7)	33	13 (39.4)	16 (11)
H10	Rimini	70,307	53,728 (75.1)	61	41 (67.2)	40 (37)
H11 *	Piacenza	35,183	29,865 (84.9)	10	2 (20.0)	3 (1)
H12	Piacenza	1341	1220 (91.0)	10	3 (30.0)	0 (0)

^1^ Samples provided by the same beekeeper are marked with the same symbol (* or °). ^2^ Reads coding for valid alleles (see quality checks described in Section 2.4.2). Percentage is relative to the analyzed sample over the number of all reads obtained for that sample. ^3^ Number of valid alleles determined after the application of the filtering criteria reported in Section 2.4.2. ^4^ Number of *csd* protein alleles identified only in that sample. The percentage is over the total number of *csd* protein alleles obtained for that sample. ^5^ Number of new *csd* protein alleles. The number of private alleles that were also new is reported within brackets.

**Table 2:** Most abundant *csd* alleles (>5%) identified in the analyzed honey samples. Data are sorted by sample ID and by relative abundance.

Sample ID ^1^	*csd* Protein Allele Sequence ^2^	Abundance %	Novel ^3^
H1	KIISSLSKNTIHNNNYKYNYNNNNNYNNNYKKLQYYNINYIEQIP	53.4	
H1	KIISSLSNKTIHNNNNYKKLYYNINYIEQIP	9.7	
H1	KIISSLSNNYNYSNYNNYNNNYNNYKKLYYNINYIEQIP ^‡^	8.9	
H2 °	KIISSLSNSCNYSNNYYNKKLYYNIINIEQIP ^†^	53.7	
H2 °	KIISSLSNNYNYSNYNNYNNNYNNYKKLYYNINYIEQIP ^‡^	30.2	
H2 °	KIISSLSNKTIHNNNNYKPYYNINYIEQIP **	7.7	
H3	KIISSLSNNYKYSNYNNYNNYNNKKLYYNIINIEQIP	40.0	Yes
H3	KIISSLSNKTIHNNNNYNNYKKLYYNIINIEQIP	12.4	Yes
H3	KIISSLSNKTIHNNNYKYNYNNNNNYKKLQYYNIINIEQIP	6.3	
H4 °	KIISSLSNKTIHNNNNYKPYYNINYIEQIP **	83.6	
H5 °	KIISSLSSNYNSNNYNNYNNYKQLCYNINYIEQIP ^@^	39.6	
H5 °	KIISSLSNNYKYSNYNNYNNYNKKLYYKNYIINIEQIP	12.0	
H5 °	KIISSLSNNYNYNNKYNYNNNYNKKLYYNIINIEQIP ^§^	8.5	
H6	KIISSLSNKTIHNNNNYKPYYNINYIEQIP **	93.1	
H7	KIISSLSNNYNYNNKYNYNNNYNKKLYYNIINIEQIP ^§^	38.2	
H7	KIISSLSNKTIHNNNKYNYNNNYNNNCKKLYYNINYIEQIP	8.9	Yes
H8	KIISSLSNKTIHNNNNYKPYYNINYIEQIP **	33.3	
H8	KIISSLSSNYNSNNYNNYNNYKQLCYNINYIEQIP ^@^	25.9	
H8	KITSSLSNNYNSNNYNKYNYNNSKKLYYNINYIEQIP	13.2	
H8	KIISSLSNKTIHNNNNYKYNYNNNNYKNYNNYKKLYYNINYIEQIP	5.8	
H9 *	KIISSLSNKTIHNNNNYKYNYNNNNYKPYYNINYIEQIP	45.0	
H9 *	KIISSLSNKTIHNNNNYKYNYNNNYNNNNYSKKLYYNINYIEQIP	10.3	Yes
H9 *	KIISSLSNNYISNISNYNNNNNSKKLYYNINYIEQIP	5.2	
H10	KIISSLSNSCNYSNNYYNKKLYYNIINIEQIP ^†^	17.6	
H11 *	KIISSLSSNYNSNNYNNYNNYKQLCYNINYIEQIP ^@^	43.8	
H11 *	KIISSLSNKTIHNNNNYKPYYNINYIEQIP **	32.5	
H11 *	KIISSLSNNYNYNNKYNYNNNYNKKLYYNIINIEQIP ^§^	6.7	
H12	KITSSLSNNYNSNSYNNYNNNYKKLQYYNIINIEQIP	37.5	
H12	KIISSLSNNYNYSNYNNYNNYNNNYNNYNNNYNNYKKLYYNINYIEQIP	31.5	
H12	KIISSLSNKTIHNNNNYKYNYNNNNYNNNNYNNNYNNNCKKLYYNINYIEQIP	5.4	
H12	KIISSLSNNYKYSNYNNYNNYNNNSKKLYKNYIINIEQIP	5.3	
H12	KIISSLSNSCNYSNNYYNKKLYYNIINIEQIP ^†^	5.1	

^1^ Samples provided by the same beekeeper are marked with the same symbol (* or °). ^2^ The same alleles identified in different samples are marked with the same symbol (^‡,†,^**^, @^ and ^§^). ^3^ Alleles that were not present in NCBI Database (August 2021). Details are reported in Tables S1 and S2.

## Data Availability

Sequencing data covering the trimmed HVR as well as the set of 160 *csd* alleles are available in the EMBL-EBI European Nucleotide Archive (ENA) repository (http://www.ebi.ac.uk/ena; accessed on 23 September 2021) under the study accession PRJEB47528.

## Supplementary Materials

The following are available online at https://www.mdpi.com/article/10.3390/insects12100868/s1: Table S1: *csd* alleles detected in the analyzed honey samples; Table S2: Non-identical *csd* alleles detected in the analyzed honey samples; Table S3: Diversity of the *csd* alleles. Statistics are presented within sample; Table S4: Similarity between samples measured using the Jaccard index; Figure S1: Distribution of the 160 *csd* alleles across the honey samples (presence: green; absence: white); Figure S2: Rarefaction curves obtained for the twelve honey samples; Figure S3: Multiple sequence alignment of the 160 *csd* alleles.
