# Supplementary material for: Application of Next Generation Semiconductor-Based Sequencing for the Identification of Apis mellifera Complementary Sex Determiner (csd) Alleles from Honey DNA
Source: Insects. 2021 Sep 24;12(10):868. doi: 10.3390/insects12100868 (PMC8536997; doi:10.3390/insects12100868)

insects

MDPI

Supplementary Material

Application of Next Generation Semiconductor-based Sequencing for the Identification of *Apis mellifera Complementary Sex Determiner* (*csd*) Alleles from Honey DNA

Samuele Bovo, Anisa Ribani, Valerio Joe Utzeri, Valeria Taurisano, Giuseppina Schiavo, Matteo Bolner and Luca Fontanesi

Table S1. *csd* alleles detected in the analyzed honey samples. Data are sorted by sample ID and by relative abundance.

**Sample ID¹**	***csd* Protein Allele**	**No. of Reads**	**Abundance %**	**Novel Allele**	**NCBI Identifiers**
H1	KIISSLSKNTIHNNNYKYNYNNNNNYNNNYKKLQYYNINYIEQIP	92458	53.424	NO	CCF23518.1
H1	KIISSLSNKTIHNNNNYKKLYYNINYIEQIP	16867	9.746	NO	CCF23466.1, ART88609.1
H1	KIISSLSNNYNYSNYNNYNNNYNNYKKLYYNINYIEQIP	15442	8.923	NO	ABV56215.1
H1	KIISSLSNSCNYSNNYYNKKLYYNIINIEQIP	7141	4.126	NO	CCF23469.1, ART88611.1
H1	KITSSLSNNYNSNSYNNYNNNYKKLQYYNIINIEQIP	5955	3.441	NO	QEN96041.1, CCF23480.1
H1	KIISSLSNNYNYNNKYNYNNNYNKKLYYNIINIEQIP	5628	3.252	NO	AEI99749.1, ABV56221.1
H1	KIISSLSNKTIHNNNNYKYNYNNNCKKLYYNINYIEQIP	4500	2.600	NO	QEN96024.1, ABD14096.1
H1	KIISSLSNNYNYSNYNNYNNNNNYNNNNYNYKKLYYNINYIEQIP	526	0.304	YES	
H1	KIISSLSNKTIHNNNNYKYNYNNNNYNNNCKKLYYNINYIEQIP	331	0.191	NO	ABD14092.1, ART88599.1
H1	KIISSLSKNTIHNNNYKYNYNNNNYYNNNYKKLQYYNINYIEQIP	198	0.114	YES	
H1	KIISSLYENTIHNNNYKYNYNNNNNYNNNYKKLQYYNINYIEQIP	168	0.097	YES	
H1	KIISSLSKNTIHNNNYKYNYNNNNYNNNYKKLQYYNINYIEQIP	118	0.068	YES	
H1	KIISSLSKNTIHNNNYKYNYNNNNNNNNNYKKLQYYNINYIEQIP	67	0.039	YES	
H1	KIISSLSKNTIHNNNYKYNYYNNNNYNNNYKKLQYYNINYIEQIP	41	0.024	YES	
H1	KIISSLSKNTIHNNNYKYNYNNNNNYNNNYKNYNNYNINYIEQIP	29	0.017	YES	
H1	KIISSLSNNYKYSNYNNYNNYNNKKLYYNIINIEQIP	18	0.010	YES	
H1	KIISSLSNKTIHNNNNYNNYKKLYYNIINIEQIP	11	0.006	YES	
H2°	KIISSLSNSCNYSNNYYNKKLYYNIINIEQIP	40552	53.744	NO	CCF23469.1, ART88611.1
H2°	KIISSLSNNYNYSNYNNYNNNYNNYKKLYYNINYIEQIP	22816	30.238	NO	ABV56215.1
H2°	KIISSLSNKTIHNNNNYKPYYNINYIEQIP	5786	7.668	NO	AGA84534.1, ADJ57956.1

H2°	KIISSLSNNYNYNNNNYNNNYNKKLYYNINYIEQIP	1185	1.570	NO	AEI99714.1, AEI99759.1, AEI99724.1, AEI99744.1, AEI99738.1, ADJ57960.1
H2°	KIISSLSSNYNSNNYNNYNNYKQLCYNINYIEQIP	655	0.868	NO	ART88596.1, CCF23477.1
H2°	KIISSLSNKTIHNNNNYKYNYNNNNYNNNNYNNNYNNNCKKLYYN-INYIEQIP	434	0.575	NO	ART88514.1
H2°	KIISSLSNNYNYNNYNNNYKPLYYNINYIEQIP	342	0.453	NO	AGA84527.1, AEI59463.1, ABD14105.1, AAS86660.1, AAS86659.1, AAS86661.1
H2°	KIISSLSNNYNYNNCNYKHKLYYNIINIEQIP	162	0.215	NO	ADJ57962.1
H2°	KIISSLSNNYNYGNYNNYNNNYNNYKKLYYNINYIEQIP	23	0.030	YES	
H2°	KIISSLSNKTIHNNNYKPYYNINYIEQIP	19	0.025	YES	
H2°	KIISSLSNSCNYSNNYYNKNYINNIINIEQIP	16	0.021	YES	
H2°	KIISSLSNSCNYSNNYYNKKLYYNIVNIEQIP	13	0.017	YES	
H2°	KIISSLSNKTIHNNNNYKQLCYNINYIEQIP	12	0.016	YES	
H3	KIISSLSNNYKYSNYNNYNNYNNKKLYYNIINIEQIP	165478	39.970	YES	
H3	KIISSLSNKTIHNNNNYNNYKKLYYNIINIEQIP	51476	12.434	YES	
H3	KIISSLSNKTIHNNNYKYNYNNNNNYKKLQYYNIINIEQIP	26205	6.330	NO	CCF23501.1
H3	KIISSLSNKTIHNNNNYKYNYNNNNYNNNCKKLYYNINYIEQIP	10578	2.555	NO	ABD14092.1, ART88599.1
H3	KIISSLSNSCNYSNNYYNKKLYYNIINIEQIP	2947	0.712	NO	CCF23469.1, ART88611.1
H3	KIISSLSNNYNYNNNNYNNNYNKKLYYNINYIEQIP	1671	0.404	NO	AEI99714.1, AEI99759.1, AEI99724.1, AEI99744.1, AEI99738.1, ADJ57960.1
H3	KIISSLSNNYNYSNYNNYNNNYNNYKKLYYNINYIEQIP	1630	0.394	NO	ABV56215.1
H3	KIISSLSNNYKYSNYNNYNNYNNNSKKLYKNYIINIEQIP	875	0.211	NO	QEN96020.1, ART88598.1, AQZ41187.1, AQZ41197.1, AQZ41198.1, CCF23491.1
H3	KIISSLSNKTIHNNNNYKYNYNNNCKKLYYNINYIEQIP	801	0.193	NO	QEN96024.1, ABD14096.1
H3	KIISSLSNKTIHNNNNYKNYNNYNNNNYKNYNNYKKLYYNIINIEQIP	706	0.171	NO	CCF23534.1
H3	KIISSLSNKTIHNNNNYKYNYNNNNNNYKNYNNYKKLYYNINYIEQIP	700	0.169	NO	ADJ57947.1, AGA84535.1
H3	KITSSLSNSCNYSNNYNNNYNNTKKLYYNINYIEQIP	627	0.151	NO	AEI99783.1, AGZ61869.1

H3	KIISSLSNNYNYNNYNNTNNINKQLYYNINYIEQIP	432	0.104	NO	QEN96077.1, AQZ41218.1, ABV56218.1
H3	KIISSLSNNYISNISNYNNNNNSKKLYYNINYIEQIP	372	0.090	NO	CCF23481.1, CCF23482.1, AGZ61876.1
H3	KIISSLSNNYNYNNNNYNKKLYYNINYIEQIP	300	0.072	NO	QEN96047.1, CCF23467.1
H3	KIISSLSKNTIHNNNYKYNYNNNNNYNNNYKKLQYYNINYIEQIP	292	0.071	NO	CCF23518.1
H3	KIISSLSNKTIHNNNNYNNYNNKKLYYNIINIEQIP	283	0.068	YES	
H3	KIISSLSNKTIHNNNNYKYNYNNNNYKNYNNYKKLYYNINYIEQIP	281	0.068	NO	AQZ41220.1
H3	KIISSLSNNTIHNNNYKYNYNNNYNNYKKLYYNINYIEQIP	272	0.066	NO	QEN96073.1
H3	KIISSLSNKTIHNNNYNNNNYNNNYKNYNNSKKLYYNINYIEQIP	229	0.055	NO	ADJ57946.1, AIS73043.1, ART88552.1
H3	KIISSLSNNYNYNNNNYNNYNNNYNNNYNKKLYYNIINIEQIP	194	0.047	NO	AQZ41205.1, AQZ41204.1, CCF23512.1
H3	KITSSLSNNYNSNNYNKYNYNNSKKLYYNINYIEQIP	189	0.046	NO	CCF23483.1
H3	KIISSLSNNYKYSNYNNYNNYKKLYYNIINIEQIP	178	0.043	YES	
H3	KIISSLSNKTIHNNNNYNNNNYNNYKKLYYNINYIEQIP	159	0.038	YES	
H3	KIISSLSNNYKYSNYNNYNNYNYKKLYYNIINIEQIP	140	0.034	YES	
H3	KIISSLSNNTIHNNNYKYNYNNNNYNNNYNKKLYYKNYIINIEQIP	139	0.034	NO	CCF23520.1
H3	KIISSLSNKTIHNNNYKYNYNNNNYKKLQYYNIINIEQIP	134	0.032	YES	
H3	KIISSLSNKTIHNNNYKYNYNNNNYYKKLQYYNIINIEQIP	129	0.031	YES	
H3	KIISSLSNNYKYSNYNNYNNYNNNNYNYNKKLYYKNYIINIEQIP	120	0.029	YES	
H3	KIISSLSNNYNYSNYNNYNNYNNNYNNYKKLYYNINYIEQIP	106	0.026	NO	CCF23508.1
H3	KIISSLSNNYNYNNNNYNKYNYNNYNTNYKKLYYKNYIINIEQIP	106	0.026	YES	
H3	KIISSLSNKTIHNNNNYKYNYNNNNYNNNCKNYITILINIEQIP	106	0.026	YES	
H3	KIISSLSNNTIHNNNNYNKKLYYNIINIEQIP	99	0.024	NO	CCF23470.1, AGZ61872.1, AGZ61871.1, AGZ61874.1, AGZ61866.1
H3	KIISSLSNKTIHNNNNYNNYKKLYYNINYIEQIP	94	0.023	NO	AQZ41207.1, AQZ41206.1, CCF23474.1
H3	KIISSLSNNYNYSNYNNYNNKYYNNNNYNNSKKLYYNINYIEQIP	88	0.021	NO	AIS73046.1, CCF23522.1
H3	KIISSLSNKTIHNNNYKYNYNNNNNYKKLYYNIINIEQIP	85	0.021	YES	
H3	KIISSLSNKTIHNNNNYNNKKLYYNIINIEQIP	70	0.017	YES	
H3	KIISSLSNKTIHNNNNYKYNYNNNNYNNNNYNNNYNNNCKKLYYN-INYIEQIP	21	0.005	NO	ART88514.1
H3	KIISSLSNNYISNISNYNNNNSKKLYYNINYIEQIP	14	0.003	YES	
H3	KIISSLSNKTIHNNNNYKKLQYYNIINIEQIP	12	0.003	YES	
H4°	KIISSLSNKTIHNNNNYKPYYNINYIEQIP	70461	83.577	NO	AGA84534.1, ADJ57956.1
H4°	KIISSLSSNYNSNNYNNYNNYKQLCYNINYIEQIP	10422	2.517	NO	ART88596.1, CCF23477.1
H4°	KIISSLSNKTIHNNNYYKPYYNINYIEQIP	247	0.060	YES	

H4°	KIISSLSNKTIHNNNNYNNNNYNNNNYKKLQYYNINYIEQIP	217	0.052	NO	QEN96043.1, CCF23505.1, ART88502.1
H4°	KIISSLSNKTIHNNNNNYKKLQYYNINYIEQIP	181	0.044	NO	CCF23465.1, ART88516.1
H4°	KIISSLSNKTIHNNNYKPYYNINYIEQIP	134	0.032	YES	
H4°	KIISSLSNNTIHNNNNYKYNYNNNYNNYNNKKLYYNINYIEQIP	68	0.016	YES	
H4°	KIISSLSNKTIHNYNNYKQLCYNINYIEQIP	67	0.016	YES	
H4°	KIISSLSNNYNYNNKYNYNNNYNKKLYYNIINIEQIP	64	0.015	NO	AEI99749.1, ABV56221.1
H4°	KIISSLSNHYNYNNNKYNNYNNDYKKLYYNINYIEQIP	42	0.010	NO	ADJ57943.1, AQZ41194.1
H4°	KIISSLSNNYKYSNYNNYNNNYNNYKKLYKNYIINIEQIP	27	0.007	NO	AGZ61870.1, AGZ61867.1, AGZ61873.1
H4°	KIISSLSNNYKYSNYNNYNNYNKKLYYKNYIINIEQIP	25	0.006	NO	QEN96085.1, QEN96088.1, ADJ57958.1, ABD14104.1, AGA84523.1
H4°	KIISSLSNKTIHNNNNYKQLCYNINYIEQIP	25	0.006	YES	
H4°	KIISSLSNNYNYNNNNYNNNYNKKLYYNINYIEQIP	17	0.004	NO	AEI99714.1, AEI99759.1, AEI99724.1, AEI99744.1, AEI99738.1, ADJ57960.1
H5°	KIISSLSSNYNSNNYNNYNNYKQLCYNINYIEQIP	157490	39.637	NO	CCF23477.1
H5°	KIISSLSNNYKYSNYNNYNNYNKKLYYKNYIINIEQIP	47689	12.002	NO	QEN96085.1, QEN96088.1, ADJ57958.1, ABD14104.1, AGA84523.1
H5°	KIISSLSNNYNYNNKYNYNNNYNKKLYYNIINIEQIP	33722	8.487	NO	AEI99749.1, ABV56221.1
H5°	KITSSLSNSCNYSNNYNNNYNNTKKLYYNINYIEQIP	13007	3.274	NO	AEI99783.1, AGZ61869.1
H5°	KIISSLSNKTIHNNNNYNNNNYNNYKKLYYNIINIEQIP	8659	2.179	NO	AEI99777.1
H5°	KIISSLSNNYKYSNYNNYNNNYNNYKKLYKNYIINIEQIP	8233	2.072	NO	AGZ61870.1, AGZ61867.1, AGZ61873.1
H5°	KIISSLSNNYNYNNYNNTNNINKQLYYNINYIEQIP	5503	1.385	NO	QEN96077.1, AQZ41218.1, ABV56218.1
H5°	KIISSLSNKTIHNNNNYKYNYNNNNYKKLQYYNINYIEQIP	4931	1.241	NO	AGA84528.1
H5°	KIISSLSNKTIHNNNNYKYNYNNNNYNNNYNNNCKKLYYNIINIEQIP	4571	1.150	NO	QEN96035.1, AEI99762.1, ART88521.1
H5°	KIISSLSNKTIHNNNNYNNYKKLYYNINYIEQIP	4141	1.042	NO	AQZ41207.1, AQZ41206.1, CCF23474.1

H5°	KIISSLSNNYKYNNYNNYNNNNYNNNNYNNNSKKLYYNIINIEQIP	3569	0.898	YES	
H5°	KIISSLSNNYNSNSLLSYNNYNNNNNYNKLYYNINYIEQIP	3080	0.775	NO	CCF23506.1
H5°	KITSSLSSSCNYSNNYNNYYNNNKKLYYNINYIEQIP	738	0.186	NO	QEN96015.1
H5°	KIISSLSNKTIHNNNNYKNYNYKKLYYNIINIEQIP	620	0.156	NO	CCF23479.1
H5°	KIISSLSNNYKYSNYNNYNNYNKIILQNYIINIEQIP	251	0.063	YES	
H5°	KIISSLSNNYKYSNYNNYNNYNKNYITKINIINIEQIP	230	0.058	YES	
H5°	KIISSLSNNYKYSNYNNYNNYNKNYITKNYIINIEQIP	186	0.047	YES	
H5°	KIISSLSNKTIHNNNNYKYNYNNNYNNNSKKLYYNINYIEQIP	101	0.025	NO	AGA84537.1, CCF23514.1, ART88504.1
H5°	KIISFLSSNYNSNNYNNYNNYKQLCYNINYIEQIP	67	0.017	YES	
H5°	KIISSLSNKTIHNNNNYKPYYNINYIEQIP	16	0.004	NO	AGA84534.1, ADJ57956.1
H6	KIISSLSNKTIHNNNNYKPYYNINYIEQIP	31996	93.147	NO	AGA84534.1, ADJ57956.1
H6	KIISSLSSNYNSNNYNNYNNYKQLCYNINYIEQIP	1106	3.220	NO	ART88596.1, CCF23477.1
H6	KIISSLSNKTIHNNNNYKQLCYNINYIEQIP	95	0.277	YES	
H6	KIISSLSNKTIHNYNNYKQLCYNINYIEQIP	72	0.210	YES	
H6	KIISSLSNKTIHNNNNYKPYYNIINIEQIP	59	0.172	YES	
H6	KIISSLSNNYNSNSLLSYNNYNNNNNYNKLYYNINYIEQIP	45	0.131	NO	CCF23506.1
H6	KIISSLSNNYNYNNNNYNNYNNNYNNNYNKKLYYNIINIEQIP	40	0.116	NO	AQZ41205.1, AQZ41204.1, CCF23512.1
H6	KIISSLSNKTIHNNNYYKPYYNINYIEQIP	19	0.055	YES	
H6	KIISSLSNKTIHNNNYKPYYNINYIEQIP	18	0.052	YES	
H6	KIISSLSSNYNSNNYNNYNNYKPYYNINYIEQIP	17	0.049	YES	
H6	KIISSLSNNYNYNNKYNYNNNYNKKLYYNIINIEQIP	15	0.044	NO	AEI99749.1, ABV56221.1
H6	KITSSLSNNYNSNSYNNYNNNYKKLQYYNIINIEQIP	11	0.032	NO	QEN96041.1, CCF23480.1
H6	KIISSLSSNYNSNNYNNNNNYKPYYNINYIEQIP	10	0.029	YES	
H7	KIISSLSNNYNYNNKYNYNNNYNKKLYYNIINIEQIP	38145	38.153	NO	AEI99749.1, ABV56221.1
H7	KIISSLSNKTIHNNNKYNYNNNYNNNCKKLYYNINYIEQIP	8942	8.944	YES	
H7	KIISSLSNNYNYNNKYNYNNNYNNNCKKLYYNINYIEQIP	255	0.255	YES	
H7	KIISSLSNKTIHNNNKYNYNNNYNKKLYYNIINIEQIP	215	0.215	YES	
H7	KIISSLSNKTIHNNNNYNNYKKLYYNINYIEQIP	79	0.079	NO	AQZ41207.1, AQZ41206.1, CCF23474.1
H7	KIISSLSNKTIHNNNNYKYNYNNNNYKPYYNINYIEQIP	74	0.074	NO	AEI99745.1
H7	KIISSLSNNYNYNNKYNYNNNYNKKLYYNINYIEQIP	72	0.072	YES	
H7	KIISSLSNNYNYNNKYNYNNNYNKNYIYNIINIEQIP	70	0.070	YES	
H7	KIISSLSNKTIHNNNKYNYNNNYNNNYNKKLYYNIINIEQIP	69	0.069	YES	
H7	KIISSLSNKTIHNNNKYNYNNNYNNNCKKLYYNIINIEQIP	53	0.053	YES	
H7	KIISSLSNSCNYSNNYYNKKLYYNIINIEQIP	51	0.051	NO	CCF23469.1, ART88611.1
H7	KIISSLSNNYNYNNKYNYNNNYNKNYINNIINIEQIP	39	0.039	YES	

H7	KIISSLSNKTIHNNNNNYKPYYNINYIEQIP	39	0.039	NO	AGA84534.1, ADJ57956.1
H7	KIISSLSNNYNYNNKYNYNNNCKKLYYNINYIEQIP	34	0.034	YES	
H7	KIISSLSSNYNSNNYNNYNNYKQLCYNINYIEQIP	30	0.030	NO	ART88596.1, CCF23477.1
H7	KIISYLSNNYNYNNKYNYNNNYNKKLYYNIINIEQIP	29	0.029	YES	
H7	KITSSLSNSCNYSNNYNNNYNNTKKLYYNINYIEQIP	28	0.028	NO	AEI99783.1, AGZ61869.1
H7	KITSSLSNNYNSNSYNNYNNNYKKLQYYNIINIEQIP	26	0.026	NO	QEN96041.1, CCF23480.1
H7	KIISSLSNNYNYNIKYNYNNNYNKKLYYNIINIEQIP	17	0.017	YES	
H7	KIISSLSNNYNYNNNYNYNNNYNKNYITNIINIEQIP	17	0.017	YES	
H7	KIISSLSNKTIHNNNNNYKYNYNNNYNNNNYSKKLYYNINYIEQIP	15	0.015	YES	
H7	KIISSLSNNYNYNNNNYNNNYNKKLYYNINYIEQIP	12	0.012	NO	AEI99714.1, AEI99759.1, AEI99724.1, AEI99744.1, AEI99738.1, ADJ57960.1
H7	KIISSLSNNYISNISNYNNNNNSKKLYYNINYIEQIP	10	0.010	NO	CCF23481.1, CCF23482.1, AGZ61876.1
H8	KIISSLSNKTIHNNNNNYKPYYNINYIEQIP	13913	33.332	NO	AGA84534.1, ADJ57956.1
H8	KIISSLSSNYNSNNYNNYNNYKQLCYNINYIEQIP	10814	25.907	NO	ART88596.1, CCF23477.1
H8	KITSSLSNNYNSNNYNKYNYNNSKKLYYNINYIEQIP	5520	13.224	NO	CCF23483.1
H8	KIISSLSNKTIHNNNNNYKYNYNNNNYKNYNNYKKLYYNINYIEQIP	2419	5.795	NO	AQZ41220.1
H8	KIISSLSNKTIHNNNNNYKYNYNNNCKKLYYNINYIEQIP	1963	4.703	NO	QEN96024.1, ABD14096.1
H8	KIISSLSSNYNSNNYNNYSTNYKQLQYCYNINYIEQIP	728	1.744	NO	QEN96023.1, AEI99730.1, AEI99775.1, AEI99771.1, AEI99764.1, AEI99731.1, AEI99751.1, AEI99752.1, AEI99725.1
H8	KIISSLSNNYNYNNCNYKHKLYYNIINIEQIP	591	1.416	NO	ADJ57962.1
H8	KIISSLSNNYNYSNYNNNNNNYNNYKQLCYNINYIEQIP	515	1.234	NO	AIS73044.1, CCF23488.1, CCF23489.1
H8	KITSSLSNSCNYSNNYNNNYNNTKKLYYNINYIEQIP	355	0.850	NO	AEI99783.1, AGZ61869.1
H8	KIISSLSNNYISNISNYNNNNNSKKLYYNINYIEQIP	198	0.474	NO	CCF23481.1, CCF23482.1, AGZ61876.1
H8	KIISSLSNNYKYSNYNNYNNYNNKKLYYNIINIEQIP	191	0.458	YES	
H8	KIISSLSNKTIHNNNNNYKYNYNNNYNNNHYNNNYKKLQYYNI-INIEQIP	84	0.201	NO	AIS73038.1, AQZ41201.1

H8	KIISSLSNNYNYNNKYNYNNNYNKKLYYNIINIEQIP	45	0.108	NO	AEI99749.1, ABV56221.1
H8	KIISSLSNKTIHNYNNYKQLCYNINYIEQIP	32	0.077	YES	
H8	KIISSLSNKTIHNNNNYKYNYNNNNYKPYYNINYIEQIP	32	0.077	NO	AEI99745.1
H8	KIISSLSNSCNYSNNYYNKKLYYNIINIEQIP	21	0.050	NO	CCF23469.1, ART88611.1
H8	KIISSLSNNYNYSNYNNNNNYNNYKQLCYNINYIEQIP	10	0.024	YES	
H9*	KIISSLSNKTIHNNNNYKYNYNNNNYKPYYNINYIEQIP	43968	44.987	NO	AEI99745.1
H9*	KIISSLSNKTIHNNNNYKYNYNNNNYNNNNYSKKLYYNINYIEQIP	10107	10.341	YES	
H9*	KIISSLSNNYISNISNYNNNNNSKKLYYNINYIEQIP	5113	5.231	NO	CCF23481.1, CCF23482.1, AGZ61876.1
H9*	KIISSLSNKTIHNNNNYKYNYNNNNNNNYKNYNNYKKLYYNINYIEQIP	2050	2.098	NO	ADJ57947.1, AGA84535.1
H9*	KIISSLSSNYNSNNYNNYNNYKQLCYNINYIEQIP	1803	1.845	NO	ART88596.1, CCF23477.1
H9*	KIISSLSNKTIHNNNNYKPYYNINYIEQIP	1776	1.817	NO	AGA84534.1, ADJ57956.1
H9*	KITSSLSNNYNSNSYNNNYNNNYYNKKLQYYNINYIEQIP	1055	1.079	NO	CCF23486.1
H9*	KIISSLSNNTIHNNNNYKYNYNNNYNNYNNKKLYYNINYIEQIP	1055	1.079	YES	
H9*	KIISSLSNNYKYSNYNNNYNNYNKKLYYKNYIINIEQIP	837	0.856	NO	QEN96085.1, QEN96088.1, ADJ57958.1, ABD14104.1, AGA84523.1
H9*	KIISSLSNNYKYSNYNNNNYNNNSKKLYYNINYIEQIP	796	0.814	NO	AQZ41199.1, CCF23485.1
H9*	KIISSLSNNYKYSNYNNYNNYNNNSKKLYKNYIINIEQIP	756	0.774	NO	QEN96020.1, ART88598.1, AQZ41187.1, AQZ41197.1, AQZ41198.1, CCF23491.1
H9*	KIISSLSNKTIHNNNNYKYNYNNNNYKTILTILINIEQIP	744	0.761	YES	
H9*	KIISSLSNNYNYSNYNNYNNKYYNNNNYNNSKKLYYNINYIEQIP	729	0.746	NO	AIS73046.1, CCF23522.1
H9*	KIISSLSNNYNYNNKYNYNNNYNKKLYYNIINIEQIP	677	0.693	NO	AEI99749.1, ABV56221.1
H9*	KIISSLSNHYNYNNNKYNNYNNDYKKLYYNINYIEQIP	550	0.563	NO	ADJ57943.1, AQZ41194.1
H9*	KIISSLSNKTIHNNNNYKYNYNNNYNNNSKKLYYNINYIEQIP	508	0.520	NO	AGA84537.1, CCF23514.1, ART88504.1
H9*	KIISSLSNNTIHNNNYKYNYNNNYNNYNNYKKLYYNINYIEQIP	273	0.279	YES	
H9*	KIISSLSNNYISNISNYNNNNSKKLYYNINYIEQIP	184	0.188	YES	
H9*	KIISSLSNNTIHNNNYKYNYNNNNYNNNYNKKLYYKNYIINIEQIP	149	0.152	NO	CCF23520.1
H9*	KIISSLSNNYNYNNNNYNNYNNNYNNNYNKKLYYNIINIEQIP	139	0.142	NO	AQZ41205.1, AQZ41204.1, CCF23512.1
H9*	KIISSLSNKTIHNNNNYKYNYNNNYKPYYNINYIEQIP	125	0.128	YES	
H9*	KIISSLSNKTIHNNNNYKYNYNNNYYKPYYNINYIEQIP	107	0.109	YES	

H9*	KIISSLSNNYNYNNNNYNNYNNYNNYNNNYNNNYNKKLYYN-INYIEQIP	95	0.097	YES	
H9*	KIISSLSNKTIHNNNYYKYNYNNNNYKPYYNINYIEQIP	68	0.070	YES	
H9*	KIISSLSNKTIHNNNYKYNYNNNNYKPYYNINYIEQIP	60	0.061	YES	
H9*	KIISSLSNRTIHNNNNYKYNYNNNNYKPYYNINYIEQIP	48	0.049	YES	
H9*	KIISSLSNSCNYSNNYYNKKLYYNIINIEQIP	47	0.048	NO	CCF23469.1, ART88611.1
H9*	KIISSYRTITFSNISNYNNNNNSKKLYYNINYIEQIP	40	0.041	YES	
H9*	KIISSLSNNYKYSNYNNYNNYNNNSKKLYKIILINIEQIP	34	0.035	YES	
H9*	KIISSLSNKTIHNNNNYKYNYNNNYNNNYSKKLYYNINYIEQIP	20	0.020	YES	
H9*	KIISSLSNNYNYNNNNYNNNYNKKLYYNINYIEQIP	18	0.018	NO	AEI99714.1, AEI99759.1, AEI99724.1, AEI99744.1, AEI99738.1, ADJ57960.1
H9*	KITSSLSNNYNSNSYNNYNNNYKKLQYYNIINIEQIP	16	0.016	NO	QEN96041.1, CCF23480.1
H9*	KIISSLSNNYKYSNYNNYNNYNKIILQNYIINIEQIP	11	0.011	YES	
H10	KIISSLSNSCNYSNNYYNKKLYYNIINIEQIP	29702	17.642	NO	CCF23469.1, ART88611.1
H10	KIISSLSNNYNYNNNNYNNNYNKKLYYNINYIEQIP	6623	3.934	NO	AEI99714.1, AEI99759.1, AEI99724.1, AEI99744.1, AEI99738.1, ADJ57960.1
H10	KITSSLSNNYNSNSYNNYNNNYKKLQYYNIINIEQIP	6016	3.573	NO	QEN96041.1, CCF23480.1
H10	KIISSLSNNYSYNNYNNNNYNKKLYYNINYIEQIP	2291	1.361	NO	QEN96068.1, CCF23476.1
H10	KIISSLSNKTIHNNNNYNNNNNNYNNYNNYKKLYYNVINIEQIP	1466	0.871	NO	QEN96053.1, CCF23519.1
H10	KIISSLSNKTIHNNNYKYNYNNNNNYKKLQYYNIINIEQIP	715	0.425	NO	CCF23501.1
H10	KIISSLSNSCNYSNNNYNKKLYYNINYIEQIP	698	0.415	YES	
H10	KIISSLSNNYNYNNNNYNNNYKKLQYYNIINIEQIP	601	0.357	YES	
H10	KIISSLSNKTIHNNNNYKPYYNINYIEQIP	514	0.305	NO	AGA84534.1, ADJ57956.1
H10	KIISSLSSNYNSNNYNNYNNYKQLCYNINYIEQIP	470	0.279	NO	ART88596.1, CCF23477.1
H10	KIISSLSNNYNYSNYNNYNNKYYNNNNYNNSKKLYYNINYIEQIP	382	0.227	NO	AIS73046.1, CCF23522.1
H10	KIISSLSNSCNYSNNYYNKKLYYNINYIEQIP	367	0.218	YES	
H10	KITSSLSNNYNSNSYNNYNNNYNKKLYYNINYIEQIP	333	0.198	YES	
H10	KIISSLSNNYNYNNNNYNNSKKLYYNINYIEQIP	231	0.137	YES	
H10	KIISSLSNNYSYNNYNNNYKKLQYYNIINIEQIP	190	0.113	YES	
H10	KITSSLSNNYNSNSYNNYNNYKKLYYNVINIEQIP	131	0.078	YES	
H10	KIISSLSNNYNYNNNNYNNYKKLYYNVINIEQIP	108	0.064	YES	
H10	KIISSLSNKTIHNNNNYKYNYNNNNYKPYYNINYIEQIP	94	0.056	NO	AEI99745.1
H10	KIISSLSNNYNYNNNNYNNYNNYKKLYYNVINIEQIP	93	0.055	YES	

H10	KIISSLSNNYNYNNNNYNNNYNKKLYYNIINIEQIP	90	0.053	YES	
H10	KIISSLSNSCNYSNNYNNYKKLYYNVINIEQIP	89	0.053	YES	
H10	KIISSLSNNYKYSNYNNYNNYNNNSKKLYKNYIINIEQIP	89	0.053	NO	QEN96020.1, ART88598.1, AQZ41187.1, AQZ41197.1, AQZ41198.1, CCF23491.1
H10	KIISSLSNNYSYNNYNNNYNKKLYYNINYIEQIP	88	0.052	YES	
H10	KITSSLSNNYNSNSYNNYNKKLYYNINYIEQIP	76	0.045	YES	
H10	KIISSLSNNYSYNNYNNNNYNNSKKLYYNINYIEQIP	70	0.042	YES	
H10	KIISSLSNKTIHNNNNYNNNNNNYNNYNNNYKKLQYYNIINIEQIP	62	0.037	YES	
H10	KIISYLSNSCNYSNNYYNKKLYYNIINIEQIP	59	0.035	NO	ART88611.1
H10	KIISSLSNSCNYSNNYNKKLYYNINYIEQIP	58	0.034	YES	
H10	KIISSLSNSCNYSNNYNNSKKLYYNINYIEQIP	54	0.032	YES	
H10	KIISSLSNNYSYNNYNNYKKLYYNVINIEQIP	51	0.030	YES	
H10	KIISSLSNKTIHNNNNYNKKLYYNINYIEQIP	51	0.030	YES	
H10	KITSSLSNNYNSNSYNNYNNSKKLYYNINYIEQIP	50	0.030	YES	
H10	KIISSLSNNYNYNNKYNYNNNYNKKLYYNIINIEQIP	49	0.029	NO	AEI99749.1, ABV56221.1
H10	KIISSLSNKTIHNNNNYNNNNNNYNNYNNYNKKLYYNINYIEQIP	47	0.028	YES	
H10	KIISSLSNNTIHNNNYKYNYNNKKLYYNIINIEQIP	46	0.027	NO	QEN96089.1, AQZ41203.1
H10	KIISSLSNNYSYNNYNNYNNYKKLYYNVINIEQIP	41	0.024	YES	
H10	KIISSLSNKTIHNNNNYKNYNYKKLYYNIINIEQIP	41	0.024	NO	CCF23479.1
H10	KIISSLSNKTIHNNNNYKYNYNNNNNNYKNYNNYKKLYYNINYIEQIP	41	0.024	NO	ADJ57947.1, AGA84535.1
H10	KIISSLSNNYNYNNNNYNNNYNNSKKLYYNINYIEQIP	39	0.023	YES	
H10	KIISSLSNNYSYNNYNNNNNYKKLQYYNIINIEQIP	39	0.023	YES	
H10	KIISSLSNNYSYNNYNNNNYNKKLYYNIINIEQIP	38	0.023	YES	
H10	KIISSLSNKTIHNNNNYNNNNNNYNNNYNKKLYYNINYIEQIP	36	0.021	YES	
H10	KIISSLSNNYSYNNYNNNNYKKLQYYNIINIEQIP	35	0.021	YES	
H10	KITSSLSNNYNSNSYNNYNNNNYNKKLYYNINYIEQIP	34	0.020	YES	
H10	KIISSLSNNYNYNNNNYNNNNYNKKLYYNINYIEQIP	34	0.020	YES	
H10	KIISSLSNSCNYNNNNYNKKLYYNINYIEQIP	33	0.020	YES	
H10	KIISSLSNSCNYSNNYNNNYKKLQYYNIINIEQIP	33	0.020	YES	
H10	KIISSLSNKTIHNNNNYNNNNNNYNNNNNYKKLQYYNIINIEQIP	32	0.019	YES	
H10	KIISSLSNNYNYNYNNYNNYKKLYYNVINIEQIP	31	0.018	YES	
H10	KIISSLSNNYNYNNNNYNNNNNYKKLQYYNIINIEQIP	30	0.018	YES	
H10	KIISSLSNNYISNISNYNNNNNSKKLYYNINYIEQIP	30	0.018	NO	CCF23481.1, CCF23482.1, AGZ61876.1
H10	KIISSLSNNYNYNNNNYNNNYNKNYITIFNYIEQIP	29	0.017	YES	
H10	KITSSLSNNYNSNSYNNYNNYNNYKKLYYNVINIEQIP	28	0.017	YES	
H10	KITSSLSNNYNSNSYNNNNNNYKKLQYYNIINIEQIP	28	0.017	YES	
H10	KIISSLSNKTIHNNNNYKYNYNNNYNNNNYSKKLYYNINYIEQIP	25	0.015	YES	
H10	KIISSLSNNYNYNNNNYNKKLYYNINYIEQIP	19	0.011	NO	QEN96047.1, CCF23467.1

H10	KIISSLSNKTIHNNNKYNYNNNYNNNCKKLYYNINYIEQIP	16	0.010	YES	
H10	KIISSLSNKTIHNNNNYKKLQYYNIINIEQIP	13	0.008	YES	
H10	KIISSLSNNYNYSNYNNYNNYNNNYNNYNNNYNNYKKLYYN-INYIEQIP	12	0.007	NO	CCF23536.1
H10	KITSSLSNNYNSNNYNKYNYNNSKKLYYNINYIEQIP	12	0.007	NO	CCF23483.1
H10	KITSSLSNSCNYSNNYNNNYNNTKKLYYNINYIEQIP	11	0.007	NO	AEI99783.1, AGZ61869.1
H11*	KIISSLSSNYNSNNYNNYNNYKQLCYNINYIEQIP	15394	43.754	NO	ART88596.1, CCF23477.1
H11*	KIISSLSNKTIHNNNNYKPYYNINYIEQIP	11442	32.521	NO	AGA84534.1, ADJ57956.1
H11*	KIISSLSNNYNYNNKYNYNNNYNKKLYYNIINIEQIP	2365	6.722	NO	AEI99749.1, ABV56221.1
H11*	KIISSLSNKTIHNNNNYKYNYNNNNYNNNNYNNNYNNNCKKLYYN-INYIEQIP	344	0.978	NO	ART88514.1
H11*	KIISSLSNKTIHNNNNYKYNYNNNNYNNNNYNNNNYKKLQYYN-INYIEQIP	209	0.594	NO	ADJ57954.1
H11*	KIISSLSNKTIHNNNYKPYYNINYIEQIP	30	0.085	YES	
H11*	KIISSYRAITILTIINNYNNYKQLCYNINYIEQIP	22	0.063	YES	
H11*	KIISSLSNKTIHNNNNYKYNYNNNNYKPYYNINYIEQIP	21	0.060	NO	AEI99745.1
H11*	KIISSLSNSCNYSNNYYNKKLYYNIINIEQIP	21	0.060	NO	CCF23469.1, ART88611.1
H11*	KIISFLSSNYNSNNYNNYNNYKQLCYNINYIEQIP	17	0.048	YES	
H12	KITSSLSNNYNSNSYNNYNNNYKKLQYYNIINIEQIP	503	37.509	NO	QEN96041.1, CCF23480.1
H12	KIISSLSNNYNYSNYNNYNNYNNNYNNYNNNYNNYKKLYYN-INYIEQIP	422	31.469	NO	CCF23536.1
H12	KIISSLSNKTIHNNNNYKYNYNNNNYNNNNYNNNYNNNCKKLYYN-INYIEQIP	72	5.369	NO	ART88514.1
H12	KIISSLSNNYKYSNYNNYNNYNNNSKKLYKNYIINIEQIP	71	5.295	NO	QEN96020.1, ART88598.1, AQZ41187.1, AQZ41197.1, AQZ41198.1, CCF23491.1
H12	KIISSLSNSCNYSNNYYNKKLYYNIINIEQIP	69	5.145	NO	CCF23469.1, ART88611.1
H12	KIISSLSNNYNYNNCNYKHNKLYYNIINIEQIP	38	2.834	NO	AEI99715.1, AEI99779.1, ABV56217.1
H12	KIISSLSNNYNYSNYNNYNNNNNYNNYKKLYYNINYIEQIP	14	1.044	NO	ABD14109.1, ABD14115.1, AQZ41223.1, QEN96102.1
H12	KIISSLSNNYNYNNYNKKLYYKNYIINIEQIP	11	0.820	NO	CCF23462.1
H12	KIISSLSNKTIHNNNNYKYNYNNNNYKPYYNINYIEQIP	10	0.746	NO	AEI99745.1
H12	KITSSLSNSCNYSNNYNNNYNNTKKLYYNINYIEQIP	10	0.746	NO	AEI99783.1, AGZ61869.1

[1]Samples provided by the same beekeeper have been marked with the same symbol (* or °).

Table S2. Non-identical *csd* protein alleles detected in the analyzed honey samples

csd Protein Allele	No. of Samples with the Allele	Samples with the Allele[1]	Min. Abundance (%) Detected in the Dataset	Max. Abundance (%) Detected in the Dataset	Novel Allele	NCBI Identifiers
KIISSLSKNTIHNNNYKYNYNNNNNNNNNNYKKLQYYNINYIEQIP	1	H1	0.039	0.039	Yes	
KIISSLSNKTIHNNNNYKYNYNNNNYKKLQYYNINYIEQIP	1	H5°	1.241	1.241	No	AGA84528.1
KIISSLSNKTIHNNNNYKYNYNNNNYNNNYNNNCKKLYYNIINIEQIP	1	H5°	1.150	1.150	No	QEN96035.1, AEI99762.1, ART88521.1
KIISSLSNKTIHNNNNYNNNNYNNYKKLYYNIINIEQIP	1	H5°	2.179	2.179	No	AEI99777.1
KIISSLSNNYKYNNYNNYNNNNYNNNNYNNNSKKLYYNIINIEQIP	1	H5°	0.898	0.898	Yes	
KIISSLSNNYKYSNYNNYNNYNKNYITKINIINIEQIP	1	H5°	0.058	0.058	Yes	
KIISSLSNNYKYSNYNNYNNYNKNYITKNYIINIEQIP	1	H5°	0.047	0.047	Yes	
KITSSLSSSCNYSNNYNNYYNNNKKLYYNINYIEQIP	1	H5°	0.186	0.186	No	QEN96015.1
KIISSLSNKTIHNNNNNYKKLQYYNINYIEQIP	1	H4°	0.044	0.044	No	CCF23465.1, ART88516.1
KIISSLSNKTIHNNNNYNNNNYNNNNYKKLQYYNINYIEQIP	1	H4°	0.052	0.052	No	QEN96043.1, CCF23505.1, ART88502.1
KIISSLSNNYNYGNYNNYNNNYNNYKKLYYNINYIEQIP	1	H2°	0.030	0.030	Yes	
KIISSLSNNYNYNNYNNNYKPLYYNINYIEQIP	1	H2°	0.453	0.453	No	AGA84527.1, AEI59463.1, ABD14105.1, AAS86660.1, AAS86659.1, AAS86661.1
KIISSLSNSCNYSNNYYNKKLYYNIVNIEQIP	1	H2°	0.017	0.017	Yes	
KIISSLSNSCNYSNNYYNKNYINNIINIEQIP	1	H2°	0.021	0.021	Yes	
KIISSLSNKTIHNNNNYKYNYNNNYNNNHYNNNYKKLQYYNIINIEQIP	1	H8	0.201	0.201	No	AIS73038.1, AQZ41201.1
KIISSLSNNYNYSNYNNNNNNYNNYKQLCYNINYIEQIP	1	H8	1.234	1.234	No	AIS73044.1, CCF23488.1, CCF23489.1
KIISSLSNNYNYSNYNNNNNYNNYKQLCYNINYIEQIP	1	H8	0.024	0.024	Yes	
KIISSLSSNYNSNNYNNYSTNYKQLQYCYNINYIEQIP	1	H8	1.744	1.744	No	QEN96023.1, AEI99730.1, AEI99775.1, AEI99771.1, AEI99764.1, AEI99731.1, AEI99751.1, AEI99752.1, AEI99725.1
KIISSLSNKTIHNNNNYKPYYNIINIEQIP	1	H6	0.172	0.172	Yes	
KIISSLSSNYNSNNYNNNNNYKPYYNINYIEQIP	1	H6	0.029	0.029	Yes	

KIISSLSSNYNSNNYNNYNNYKPYYNINYIEQIP	1	H6	0.049	0.049	Yes	
KIISSLSNKTIHNNNNYKNYNNYNNNNYKNYNNYKKLYYNIINIEQIP	1	H3	0.171	0.171	No	CCF23534.1
KIISSLSNKTIHNNNNYKYNYNNNNYNNNCKNYITILINIEQIP	1	H3	0.026	0.026	Yes	
KIISSLSNKTIHNNNNYNNKKLYYNIINIEQIP	1	H3	0.017	0.017	Yes	
KIISSLSNKTIHNNNNYNNNNYNNYKKLYYNINYIEQIP	1	H3	0.038	0.038	Yes	
KIISSLSNKTIHNNNNYNNYNNKKLYYNIINIEQIP	1	H3	0.068	0.068	Yes	
KIISSLSNKTIHNNNYKYNYNNNNNYKKLYYNIINIEQIP	1	H3	0.021	0.021	Yes	
KIISSLSNKTIHNNNYKYNYNNNNYKKLQYYNIINIEQIP	1	H3	0.032	0.032	Yes	
KIISSLSNKTIHNNNYKYNYNNNNYYKKLQYYNIINIEQIP	1	H3	0.031	0.031	Yes	
KIISSLSNKTIHNNNYNNNNYNNNYKNYNNSKKLYYNINYIEQIP	1	H3	0.055	0.055	No	ADJ57946.1, AIS73043.1, ART88552.1
KIISSLSNNTIHNNNNYNKKLYYNIINIEQIP	1	H3	0.024	0.024	No	CCF23470.1, AGZ61872.1, AGZ61871.1, AGZ61874.1, AGZ61866.1
KIISSLSNNTIHNNNYKYNYNNNYNNYKKLYYNINYIEQIP	1	H3	0.066	0.066	No	QEN96073.1
KIISSLSNNYKYSNYNNYNNYKKLYYNIINIEQIP	1	H3	0.043	0.043	Yes	
KIISSLSNNYKYSNYNNYNNYNNNNYNYNKKLYYKNYIINIEQIP	1	H3	0.029	0.029	Yes	
KIISSLSNNYKYSNYNNYNNYNYKKLYYNIINIEQIP	1	H3	0.034	0.034	Yes	
KIISSLSNNYNYNNNNYNKYNYNNYNTNYKKLYYKNYIINIEQIP	1	H3	0.026	0.026	Yes	
KIISSLSNNYNYSNYNNYNNYNNNYNNYKKLYYNINYIEQIP	1	H3	0.026	0.026	No	CCF23508.1
KIISSLSNKTIHNNNKYNYNNNYNKKLYYNIINIEQIP	1	H7	0.215	0.215	Yes	
KIISSLSNKTIHNNNKYNYNNNYNNNCKKLYYNIINIEQIP	1	H7	0.053	0.053	Yes	
KIISSLSNKTIHNNNKYNYNNNYNNNYNKKLYYNIINIEQIP	1	H7	0.069	0.069	Yes	
KIISSLSNNYNYNIKYNYNNNYNKKLYYNIINIEQIP	1	H7	0.017	0.017	Yes	
KIISSLSNNYNYNNKYNYNNNCKKLYYNINYIEQIP	1	H7	0.034	0.034	Yes	
KIISSLSNNYNYNNKYNYNNNYNKKLYYNINYIEQIP	1	H7	0.072	0.072	Yes	
KIISSLSNNYNYNNKYNYNNNYNKNYINNIINIEQIP	1	H7	0.039	0.039	Yes	
KIISSLSNNYNYNNKYNYNNNYNKNYIYNIINIEQIP	1	H7	0.070	0.070	Yes	
KIISSLSNNYNYNNKYNYNNNYNNNCKKLYYNINYIEQIP	1	H7	0.255	0.255	Yes	
KIISSLSNNYNYNNNYNYNNNYNKNYITNIINIEQIP	1	H7	0.017	0.017	Yes	
KIISYLSNNYNYNNKYNYNNNYNKKLYYNIINIEQIP	1	H7	0.029	0.029	Yes	
KIISSLSKNTIHNNNYKYNYNNNNNYNNNYKNYNNYNINYIEQIP	1	H1	0.017	0.017	Yes	
KIISSLSKNTIHNNNYKYNYNNNNYNNNYKKLQYYNINYIEQIP	1	H1	0.068	0.068	Yes	

KIISSLSKNTIHNNNYKYNYNNNNYYNNNYKKLQYYNINYIEQIP	1	H1	0.114	0.114	Yes	
KIISSLSKNTIHNNNYKYNYYNNNNYNNNYKKLQYYNINYIEQIP	1	H1	0.024	0.024	Yes	
KIISSLSNKTIHNNNNYKKLYYNINYIEQIP	1	H1	9.746	9.746	No	CCF23466.1, ART88609.1
KIISSLSNNYNYSNYNNYNNNNNYNNNNYNYKKLYYNINYIEQIP	1	H1	0.304	0.304	Yes	
KIISSLYENTIHNNNYKYNYNNNNNYNNNYKKLQYYNINYIEQIP	1	H1	0.097	0.097	Yes	
KIISSLSNNYNYNNCNYKHNKLYYNIINIEQIP	1	H12	2.834	2.834	No	AEI99715.1, AEI99779.1, ABV56217.1
KIISSLSNNYNYNNYNKKLYYKNYIINIEQIP	1	H12	0.820	0.820	No	CCF23462.1
KIISSLSNNYNYSNYNNYNNNNNYNNYKKLYYNINYIEQIP	1	H12	1.044	1.044	No	ABD14109.1, ABD14115.1, AQZ41223.1, QEN96102.1
KIISSLSNKTIHNNNNYNKKLYYNINYIEQIP	1	H10	0.030	0.030	Yes	
KIISSLSNKTIHNNNNYNNNNNNYNNNNNYKKLQYYNIINIEQIP	1	H10	0.019	0.019	Yes	
KIISSLSNKTIHNNNNYNNNNNNYNNNYNKKLYYNINYIEQIP	1	H10	0.021	0.021	Yes	
KIISSLSNKTIHNNNNYNNNNNNYNNYNNNYKKLQYYNIINIEQIP	1	H10	0.037	0.037	Yes	
KIISSLSNKTIHNNNNYNNNNNNYNNYNNYKKLYYNVINIEQIP	1	H10	0.871	0.871	No	QEN96053.1, CCF23519.1
KIISSLSNKTIHNNNNYNNNNNNYNNYNNYNKKLYYNINYIEQIP	1	H10	0.028	0.028	Yes	
KIISSLSNNTIHNNNYKYNYNNKKLYYNIINIEQIP	1	H10	0.027	0.027	No	QEN96089.1, AQZ41203.1
KIISSLSNNYNYNNNNYNNNNNYKKLQYYNIINIEQIP	1	H10	0.018	0.018	Yes	
KIISSLSNNYNYNNNNYNNNNYNKKLYYNINYIEQIP	1	H10	0.020	0.020	Yes	
KIISSLSNNYNYNNNNYNNNYKKLQYYNIINIEQIP	1	H10	0.357	0.357	Yes	
KIISSLSNNYNYNNNNYNNNYNKKLYYNIINIEQIP	1	H10	0.053	0.053	Yes	
KIISSLSNNYNYNNNNYNNNYNKNYITIFNYIEQIP	1	H10	0.017	0.017	Yes	
KIISSLSNNYNYNNNNYNNNYNNSKKLYYNINYIEQIP	1	H10	0.023	0.023	Yes	
KIISSLSNNYNYNNNNYNNSKKLYYNINYIEQIP	1	H10	0.137	0.137	Yes	
KIISSLSNNYNYNNNNYNNYKKLYYNVINIEQIP	1	H10	0.064	0.064	Yes	
KIISSLSNNYNYNNNNYNNYNNYKKLYYNVINIEQIP	1	H10	0.055	0.055	Yes	
KIISSLSNNYNYNYNNYNNYKKLYYNVINIEQIP	1	H10	0.018	0.018	Yes	
KIISSLSNNYSYNNYNNNNNYKKLQYYNIINIEQIP	1	H10	0.023	0.023	Yes	
KIISSLSNNYSYNNYNNNNYKKLQYYNIINIEQIP	1	H10	0.021	0.021	Yes	
KIISSLSNNYSYNNYNNNNYNKKLYYNIINIEQIP	1	H10	0.023	0.023	Yes	
KIISSLSNNYSYNNYNNNNYNKKLYYNINYIEQIP	1	H10	1.361	1.361	No	QEN96068.1, CCF23476.1
KIISSLSNNYSYNNYNNNNYNNSKKLYYNINYIEQIP	1	H10	0.042	0.042	Yes	
KIISSLSNNYSYNNYNNNYKKLQYYNIINIEQIP	1	H10	0.113	0.113	Yes	
KIISSLSNNYSYNNYNNNYNKKLYYNINYIEQIP	1	H10	0.052	0.052	Yes	
KIISSLSNNYSYNNYNNYKKLYYNVINIEQIP	1	H10	0.030	0.030	Yes	

KIISSLSNNYSYNNYNNYNNYKKLYYNVINIEQIP	1	H10	0.024	0.024	Yes	
KIISSLSNSCNYNNNNYNKKLYYNINYIEQIP	1	H10	0.020	0.020	Yes	
KIISSLSNSCNYSNNNYNKKLYYNINYIEQIP	1	H10	0.415	0.415	Yes	
KIISSLSNSCNYSNNYNKKLYYNINYIEQIP	1	H10	0.034	0.034	Yes	
KIISSLSNSCNYSNNYNNNYKKLQYYNIINIEQIP	1	H10	0.020	0.020	Yes	
KIISSLSNSCNYSNNYNNSKKLYYNINYIEQIP	1	H10	0.032	0.032	Yes	
KIISSLSNSCNYSNNYNNYKKLYYNVINIEQIP	1	H10	0.053	0.053	Yes	
KIISSLSNSCNYSNNYYNKKLYYNINYIEQIP	1	H10	0.218	0.218	Yes	
KIISYLSNSCNYSNNYYNKKLYYNIINIEQIP	1	H10	0.035	0.035	No	ART88611.1
KITSSLSNNYNSNSYNNNNNNYKKLQYYNIINIEQIP	1	H10	0.017	0.017	Yes	
KITSSLSNNYNSNSYNNYNKKLYYNINYIEQIP	1	H10	0.045	0.045	Yes	
KITSSLSNNYNSNSYNNYNNNNYNKKLYYNINYIEQIP	1	H10	0.020	0.020	Yes	
KITSSLSNNYNSNSYNNYNNNYNKKLYYNINYIEQIP	1	H10	0.198	0.198	Yes	
KITSSLSNNYNSNSYNNYNNSKKLYYNINYIEQIP	1	H10	0.030	0.030	Yes	
KITSSLSNNYNSNSYNNYNNYKKLYYNVINIEQIP	1	H10	0.078	0.078	Yes	
KITSSLSNNYNSNSYNNYNNYNNYKKLYYNVINIEQIP	1	H10	0.017	0.017	Yes	
KIISSLSNKTIHNNNNYKYNYNNNNYKTILTILINIEQIP	1	H9*	0.761	0.761	Yes	
KIISSLSNKTIHNNNNYKYNYNNNYKPYYNINYIEQIP	1	H9*	0.128	0.128	Yes	
KIISSLSNKTIHNNNNYKYNYNNNYNNNYSKKLYYNINYIEQIP	1	H9*	0.020	0.020	Yes	
KIISSLSNKTIHNNNNYKYNYNNNYYKPYYNINYIEQIP	1	H9*	0.109	0.109	Yes	
KIISSLSNKTIHNNNYKYNYNNNNYKPYYNINYIEQIP	1	H9*	0.061	0.061	Yes	
KIISSLSNKTIHNNNYYKYNYNNNNYKPYYNINYIEQIP	1	H9*	0.070	0.070	Yes	
KIISSLSNNTIHNNNYKYNYNNNYNNYNNYKKLYYNINYIEQIP	1	H9*	0.279	0.279	Yes	
KIISSLSNNYKYSNYNNNNYNNNSKKLYYNINYIEQIP	1	H9*	0.814	0.814	No	AQZ41199.1, CCF23485.1
KIISSLSNNYKYSNYNNYNNYNNNSKKLYKIILINIEQIP	1	H9*	0.035	0.035	Yes	
KIISSLSNNYNYNNNNYNNYNNYNNYNNNYNNNYNKKLYYNINYIEQIP	1	H9*	0.097	0.097	Yes	
KIISSLSNRTIHNNNNYKYNYNNNNYKPYYNINYIEQIP	1	H9*	0.049	0.049	Yes	
KIISSYRTITFSNISNYNNNNNSKKLYYNINYIEQIP	1	H9*	0.041	0.041	Yes	
KITSSLSNNYNSNSYNNNYNNNYYNKKLQYYNINYIEQIP	1	H9*	1.079	1.079	No	CCF23486.1
KIISSLSNKTIHNNNNYKYNYNNNNYNNNNYNNNNYKKLQYYNINYIEQIP	1	H11*	0.594	0.594	No	ADJ57954.1
KIISSYRAITILTIINNYNNYKQLCYNINYIEQIP	1	H11*	0.063	0.063	Yes	
KIISFLSSNYNSNNYNNYNNYKQLCYNINYIEQIP	2	H11*, H5°	0.017	0.048	Yes	
KIISSLSNKTIHNNNNYKNYNYKKLYYNIINIEQIP	2	H5°, H10	0.024	0.156	No	CCF23479.1
KIISSLSNNYKYSNYNNYNNNYNNYKKLYKNYIINIEQIP	2	H4°, H5°	0.007	2.072	No	AGZ61870.1, AGZ61867.1, AGZ61873.1
KIISSLSNNYNYNNCNYKHKLYYNIINIEQIP	2	H2°, H8	0.215	1.416	No	ADJ57962.1
KIISSLSNNYNSNSLLSYNNYNNNNNYNKLYYNINYIEQIP	2	H6, H5°	0.131	0.775	No	CCF23506.1
KIISSLSNKTIHNNNYYKPYYNINYIEQIP	2	H6, H4°	0.055	0.060	Yes	
KIISSLSNNYNYNNYNNTNNINKQLYYNINYIEQIP	2	H3, H5°	0.104	1.385	No	QEN96077.1, AQZ41218.1, ABV56218.1

KIISSLSNKTIHNNNNNYKYNYNNNNNYKNYNNNYKKLYYNINYIEQIP	2	H3, H8	0.068	5.795	No	AQZ41220.1
KIISSLSNKTIHNNNNNYKKLQYYNIINIEQIP	2	H3, H10	0.003	0.008	Yes	
KIISSLSNKTIHNNNNYKYNYNNNNNNYKKLQYYNIINIEQIP	2	H3, H10	0.425	6.330	No	CCF23501.1
KIISSLSNNYNYNNNNNYNKKLYYNINYIEQIP	2	H3, H10	0.011	0.072	No	QEN96047.1, CCF23467.1
KIISSLSNKTIHNNNNKYNYNNNNYNNNCKKLYYNINYIEQIP	2	H7, H10	0.010	8.944	Yes	
KIISSLSKNTIHNNNNYKYNYNNNNNNYNNNYKKLQYYNINYIEQIP	2	H1, H3	0.071	53.424	No	CCF23518.1
KIISSLSNKTIHNNNNNYKYNYNNNNNYNNNCKKLYYNINYIEQIP	2	H1, H3	0.191	2.555	No	ABD14092.1, ART88599.1
KIISSLSNKTIHNNNNNYNNYKKLYYNIINIEQIP	2	H1, H3	0.006	12.434	Yes	
KIISSLSNNYNYSNYNNYNNYNNNYNNYNNNNYNNYKKLYYNINYIEQIP	2	H12, H10	0.007	31.469	No	CCF23536.1
KIISSLSNKTIHNNNNNYKYNYNNNNYNNNSKKLYYNINYIEQIP	2	H9*, H5°	0.025	0.520	No	AGA84537.1, CCF23514.1, ART88504.1
KIISSLSNNYKYSNYNNYNNYNKIILQNYIINIEQIP	2	H9*, H5°	0.011	0.063	Yes	
KIISSLSNHYNYNNNKYNNYNNDYKKLYYNINYIEQIP	2	H9*, H4°	0.010	0.563	No	ADJ57943.1, AQZ41194.1
KIISSLSNNTIHNNNNNYKYNYNNNNYNNYNNKKLYYNINYIEQIP	2	H9*, H4°	0.016	1.079	Yes	
KIISSLSNNTIHNNNNYKYNYNNNNNYNNNYNKKLYYKNYIINIEQIP	2	H9*, H3	0.034	0.152	No	CCF23520.1
KIISSLSNNYISNISNYNNNNSKKLYYNINYIEQIP	2	H9*, H3	0.003	0.188	Yes	
KIISSLSNKTIHNNNNNYKQLCYNINYIEQIP	3	H2°, H4°, H6	0.006	0.277	Yes	
KIISSLSNKTIHNYNNYKQLCYNINYIEQIP	3	H6, H4°, H8	0.016	0.210	Yes	
KIISSLSNKTIHNNNNNYNNYKKLYYNINYIEQIP	3	H7, H3, H5°	0.023	1.042	No	AQZ41207.1, AQZ41206.1, CCF23474.1
KIISSLSNNYNYSNYNNYNNNYNNYKKLYYNINYIEQIP	3	H1, H2°, H3	0.394	30.238	No	ABV56215.1
KIISSLSNKTIHNNNNNYKYNYNNNCKKLYYNINYIEQIP	3	H1, H3, H8	0.193	4.703	No	QEN96024.1, ABD14096.1
KIISSLSNNYKYSNYNNYNNYNNKKLYYNIINIEQIP	3	H1, H3, H8	0.010	39.970	Yes	
KITSSLSNNYNSNNYNKYNYNNSKKLYYNINYIEQIP	3	H10, H3, H8	0.007	13.224	No	CCF23483.1
KIISSLSNNYKYSNYNNYNNYNKKLYYKNYIINIEQIP	3	H9*, H4°, H5°	0.006	12.002	No	QEN96085.1, QEN96088.1, ADJ57958.1, ABD14104.1, AGA84523.1
KIISSLSNNYNYNNNNYNNYNNNYNNNYNKKLYYNIINIEQIP	3	H9*, H6, H3	0.047	0.142	No	AQZ41205.1, AQZ41204.1, CCF23512.1
KIISSLSNKTIHNNNNNYKYNYNNNNNNNYKNYNNNYKKLYYNINYIEQIP	3	H9*, H3, H10	0.024	2.098	No	ADJ57947.1, AGA84535.1

KIISSLSNNYNYSNYNNYNNKYYNNNNYNNSKKLYYNINYIEQIP	3	H9*, H3, H10	0.021	0.746	No	AIS73046.1, CCF23522.1
KIISSLSNKTIHNNNNYKYNYNNNYNNNNYSKKLYYNINYIEQIP	3	H9*, H7, H10	0.015	10.341	Yes	
KIISSLSNKTIHNNNYKPYYNINYIEQIP	4	H2°, H11*, H4°, H6	0.025	0.085	Yes	
KIISSLSNKTIHNNNNYKYNYNNNNYNNNNYNNNYNNNCKKLYYNINYIEQIP	4	H2°, H11*, H3, H12	0.005	5.369	No	ART88514.1
KIISSLSNNYKYSNYNNYNNYNNNSKKLYKNYIINIEQIP	4	H9*, H12, H3, H10	0.053	5.295	No	QEN96020.1, ART88598.1, AQZ41187.1, AQZ41197.1, AQZ41198.1, CCF23491.1
KIISSLSNNYISNISNYNNNNNSKKLYYNINYIEQIP	5	H9*, H7, H10, H3, H8	0.010	5.231	No	CCF23481.1, CCF23482.1, AGZ61876.1
KITSSLSNSCNYSNNYNNNYNNTKKLYYNINYIEQIP	6	H7, H5°, H8, H10, H12, H3	0.007	3.274	No	AEI99783.1, AGZ61869.1
KIISSLSNKTIHNNNNYKYNYNNNNYKPYYNINYIEQIP	6	H7, H8, H9*, H10, H12, H11*	0.056	44.987	No	AEI99745.1
KITSSLSNNYNSNSYNNYNNNYKKLQYYNIINIEQIP	6	H7, H1, H6, H9*, H10, H12	0.016	37.509	No	QEN96041.1, CCF23480.1
KIISSLSNNYNYNNNNYNNNYNKKLYYNINYIEQIP	6	H7, H9*, H2°, H4°, H10, H3	0.004	3.934	No	AEI99714.1, AEI99759.1, AEI99724.1, AEI99744.1, AEI99738.1, ADJ57960.1
KIISSLSNKTIHNNNNYKPYYNINYIEQIP	9	H7, H6, H5°, H8, H9*, H2°, H4°, H10, H11*	0.004	93.147	No	AGA84534.1, ADJ57956.1
KIISSLSSNYNSNNYNNYNNYKQLCYNINYIEQIP	9	H7, H6, H5°, H8, H9*, H2°, H4°, H10, H11*	0.030	43.754	No	ART88596.1, CCF23477.1
KIISSLSNSCNYSNNYYNKKLYYNIINIEQIP	9	H7, H1, H8, H9*, H2°, H10, H12, H11*, H3	0.048	53.744	No	CCF23469.1, ART88611.1
KIISSLSNNYNYNNKYNYNNNYNKKLYYNIINIEQIP	9	H7, H1, H6, H5°, H8, H9*, H4°, H10, H11*	0.015	38.153	No	AEI99749.1, ABV56221.1

[1]Samples provided by the same beekeeper have been marked with the same symbol (* or °).

insects MDPI

Table S3. Diversity of the *csd* alleles. Statistics are presented within sample.

	Allele Length					ΔL_{HVR}[2]					$\Delta L_{HVR} + N_{SAP}$[3]				
**Sample ID[1]**	**Min**	**Max**	**Mean**	**s.d.**	**Median**	**Min**	**Max**	**Mean**	**s.d.**	**Median**	**Min**	**Max**	**Mean**	**s.d.**	**Median**
H1	31	45	40.5	5.1	44.0	0	14	5.6	4.5	6	1	24	11.5	5.3	13
H2°	29	53	34.8	6.3	32.0	0	24	5.6	5.9	3	1	25	11.4	5.5	11
H3	32	53	39.8	5.2	39.5	0	21	6.0	4.4	5	1	27	11.8	4.4	12
H4°	29	44	35.2	4.8	35.5	0	15	5.7	3.9	6	1	19	11.3	4.8	12
H5°	30	48	38.3	4.1	37.5	0	16	4.5	3.5	4	1	22	12.9	4.0	13
H6	29	43	34.0	4.5	34.0	0	13	4.8	3.6	4	1	21	10.5	5.1	11
H7	30	45	37.3	3.2	37.0	0	15	3.3	2.8	3	1	20	9.7	3.7	10
H8	30	49	37.2	4.9	37.0	0	17	5.0	4.2	5	1	22	12.1	3.9	12
H9*	30	49	39.7	4.3	39.0	0	19	4.8	3.6	4	1	24	12.9	4.8	13
H10	30	49	36.8	4.4	36.0	0	19	4.8	4.0	4	1	24	10.6	4.2	10
H11*	29	53	37.6	8.2	35.0	0	24	8.5	7.1	6	1	29	15.1	6.6	14
H12	32	53	39.3	7.0	38.0	0	21	7.4	5.4	7	6	27	14.7	5.1	14

[1]Samples provided by the same beekeeper have been marked with the same symbol ((* or °). [2]Difference in the HVR length. [3]Difference in the HVR length (ΔL_{HVR}) summed to the number of amino acid substitutions (N_{SAP}; non identical residues).

Table S4. Similarity between samples measured by using the Jaccard index.

	**H5°**	**H4°**	**H2°**	**H8**	**H6**	**H3**	**H7**	**H1**	**H12**	**H10**	**H9***	**H11***
**H5°**	1	-	-	-	-	-	-	-	-	-	-	-
**H4°**	0.172	1	-	-	-	-	-	-	-	-	-	-
**H2°**	0.065	0.227	1	-	-	-	-	-	-	-	-	-
**H8**	0.121	0.148	0.154	1	-	-	-	-	-	-	-	-
**H6**	0.138	0.350	0.182	0.154	1	-	-	-	-	-	-	-
**H3**	0.053	0.019	0.082	0.140	0.019	1	-	-	-	-	-	-
**H7**	0.132	0.121	0.125	0.212	0.125	0.086	1	-	-	-	-	-
**H1**	0.028	0.033	0.071	0.133	0.071	0.140	0.081	1	-	-	-	-
**H12**	0.034	0.000	0.095	0.125	0.045	0.087	0.138	0.080	1	-	-	-
**H10**	0.066	0.056	0.057	0.114	0.057	0.122	0.151	0.040	0.092	1	-	-
**H9***	0.128	0.175	0.095	0.136	0.122	0.141	0.191	0.064	0.103	0.146	1	-
**H11***	0.154	0.200	0.278	0.227	0.211	0.042	0.179	0.080	0.176	0.076	0.132	1

Samples provided by the same beekeeper have been marked with the same symbol (* or °).

Figure S1. Distribution of the 160 *csd* alleles across the honey samples (presence: green; absence: white).

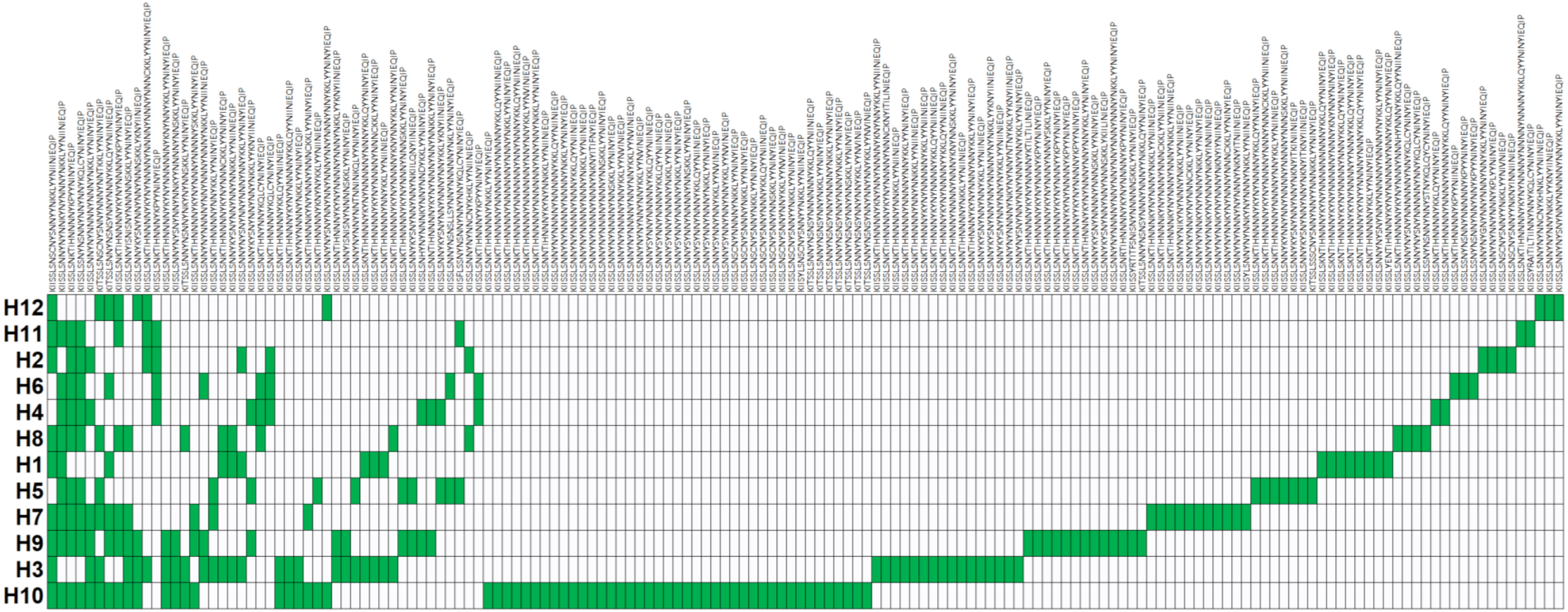

insects

Figure S2. Rarefaction curves obtained for the twelve honey samples.

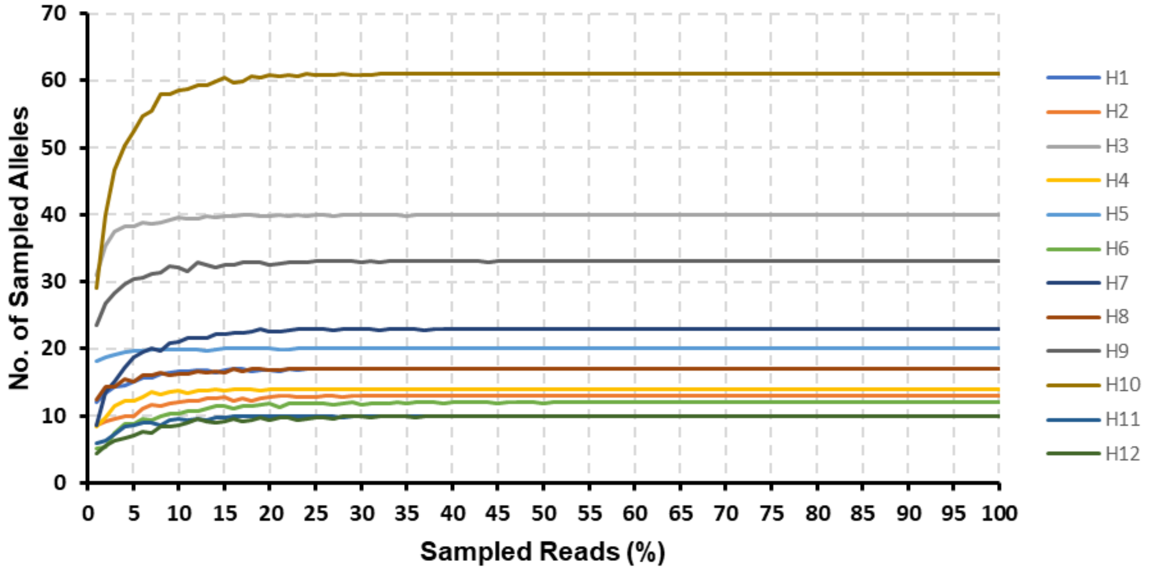

Figure S3. Multiple Sequence Alignment of the 160 *csd* protein alleles.

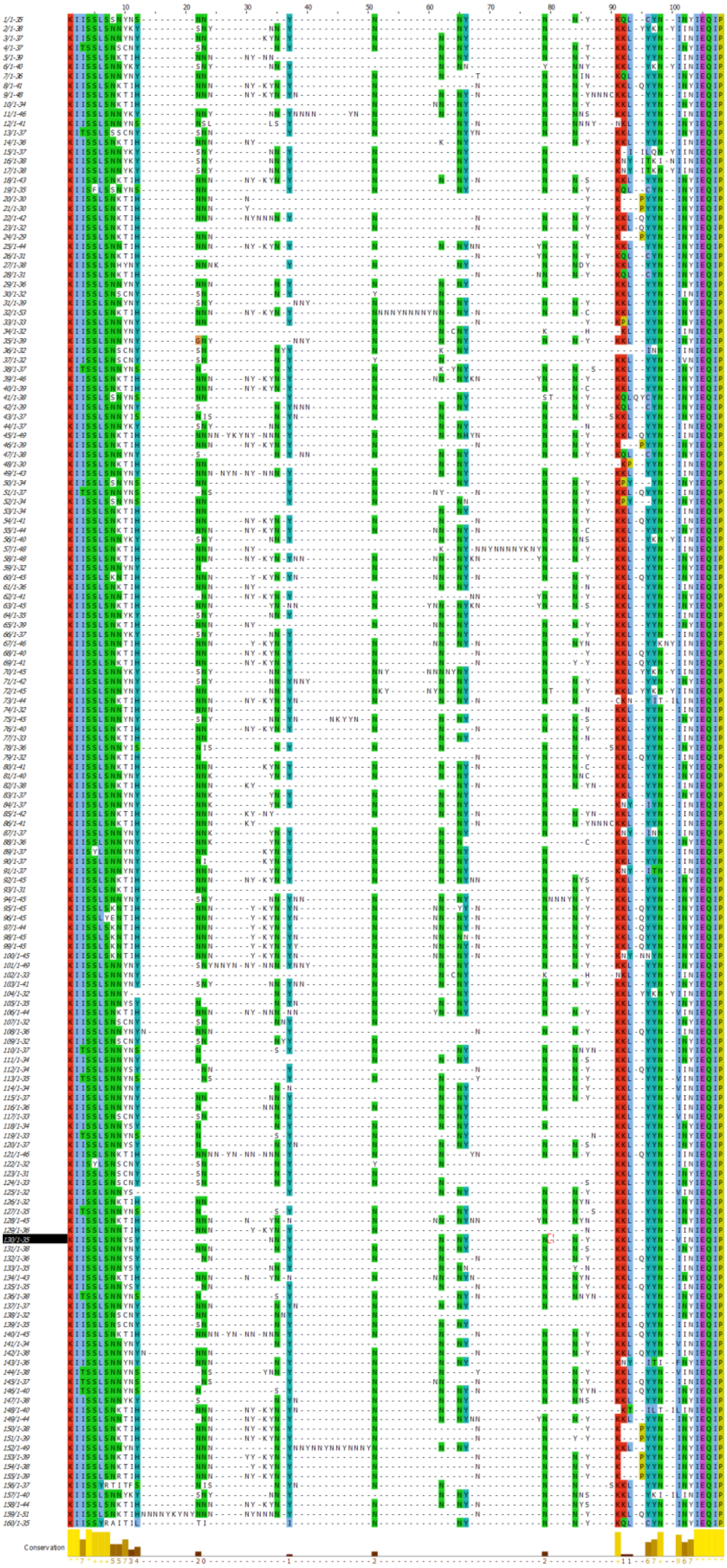

Supplement: Supplementary file 1 [file insects-12-00868-s001.zip › insects-1366701-supplementary.pdf]
